# 3D Brain Atlas Reconstructor Service—Online Repository of Three-Dimensional Models of Brain Structures

**DOI:** 10.1007/s12021-013-9199-9

**Published:** 2013-08-14

**Authors:** Piotr Majka, Jakub M. Kowalski, Natalia Chlodzinska, Daniel K. Wójcik

**Affiliations:** Nencki Institute of Experimental Biology, 3 Pasteur Street, 02-093 Warsaw, Poland

**Keywords:** Web service, Data repository, Reproducible research, Brain atlas, Tool interoperability, 3D visualization, WebGL, 3D reconstruction, Visualization

## Abstract

Brain atlases are important tools of neuroscience. Traditionally prepared in paper book format, more and more commonly they take digital form which extends their utility. To simplify work with different atlases, to lay the ground for developing universal tools which could abstract from the origin of the atlas, efforts are being made to provide common interfaces to these atlases. 3D Brain Atlas Reconstructor service (3dBARs) described here is a repository of digital representations of different brain atlases in CAF format which we recently proposed and a repository of 3D models of brain structures. A graphical front-end is provided for creating and viewing the reconstructed models as well as the underlying 2D atlas data. An application programming interface (API) facilitates programmatic access to the service contents from other websites. From a typical user’s point of view, 3dBARs offers an accessible way to mine publicly available atlasing data with a convenient browser based interface, without the need to install extra software. For a developer of services related to brain atlases, 3dBARs supplies mechanisms for enhancing functionality of other software. The policy of the service is to accept new datasets as delivered by interested parties and we work with the researchers who obtain original data to make them available to the neuroscience community at large. The functionality offered by the 3dBARs situates it at the core of present and future general atlasing services tying it strongly to the global atlasing neuroinformatics infrastructure.

**Electronic Supplementary Material:**

The online version of this article (doi:10.1007/s12021-013-9199-9) contains supplementary material, which is available to authorized users.

## Introduction

Brain atlases of various species of animals are among the most important tools and publications in neuroscience. They are used as didactic tools and in research providing neuroanatomical reference for the experimental data. Traditionally, they take the form of book publications. There exist a number of well established, high quality brain atlases of various species including rat (Swanson 2003; Paxinos and Watson 2007), mouse (Paxinos and Franklin 2008; Dong 2008), rhesus monkey (Paxinos et al. 2000), and many other, including human (e.g. DeArmond et al. 1989; Mai et al. 2007; Woolsey et al. 2007). With the advance of technology and scientific requirements, typical 2D atlases are being complemented by digital and three dimensional atlases, a specialized kind of image databases which are more flexible and better adapted to address modern conceptual, computational, analytical and visualization challenges of neuroscience. They offer various ways of presenting the data, easy navigation, searching, cross referencing, with possibilities of dynamic managing of the content (e.g. Baldock et al. 2003; Chakravarty et al. 2006; Gustafson et al. 2007; Mikula et al. 2007; Nowinski et al. 2012).

Distribution of the atlases and related data, access flexibility through both programmatic and graphical interfaces, are almost as important as the data themselves. Regardless of the form of the atlas (printed or digital), its impact and usefulness is limited if the atlas is not distributed widely enough. Scientists should have easy access to the atlases to map their data into its space and the various atlas services should be interoperable as each one usually focuses on other aspects of the brain anatomy.

The challenge of convenient and effective distribution of digital atlases and related tools has been noticed by a number of authors and institutions, for example Bjaalie (2002) and Boline et al. (2008) highlighted the need for interoperability of the atlasing tools that combine databasing, analytical and visualization capabilities in the context of mouse gene expression data. Ito (2010) analyzed technical and organizational demands for developing and maintaining digital brain atlases. MacKenzie-Graham et al. (2003, 2004) and Baldock et al. (2003) proposed frameworks for organizing and analyzing the large volumes of spatial and spatiotemporal data of the mouse brain. The Allen Institute for Brain Science (Lein et al. (2007), http://brain-map.org/) shares their vast amount of data (gene expression, brain development, connectivity) using specialized web services. Bowden and Dubach (2003) (http://braininfo.rprc.washington.edu/) provided an atlas of macaque brain based on Magnetic Resonance Imaging (MRI). There is a number of other atlases available online (honeybee brain—Brandt et al. (2005), rat hippocampus—Kjonigsen et al. (2011), human brain— http://www.thehumanbrain.info/, to name but a few); see also Ito (2010) for an overview.

A significant effort in this field has been made by the International Neuroinformatics Coordinating Facility (INCF) which has established the Program on Digital Atlasing. Its role is to make the increasingly growing collection of the rodent atlasing data widely usable to the research community (Hawrylycz et al. 2009). The Program focused initially on combining resources concerning mouse brain data (Johnson et al. 2010; Hawrylycz et al. 2011) and providing infrastructure for interconnection of various neuroanatomic data repositories (“atlasing hubs”).

Another type of software are generic atlas browsers which can handle any brain atlas provided in proper format. Stand-alone applications or web services of this type include Mouse BIRN Atlasing Toolkit (MBAT, http://mbat.loni.ucla.edu/), JatlasView (Feng et al. 2005), NeuARt II (Burns et al. 2006) or NESYS Atlas 3d (Hjornevik et al. 2007). ScalableBrainAtlas (Bakker et al. (2010), http://scalablebrainatlas.incf.org/) is an example of a fully web-based browser for brain atlases and imaging data whose functionality can be extended via a plug-in mechanism.

However, atlas browsers and web services are usually limited to viewing the data in different ways, far less frequently they provide possibility of accessing the underlying data in computable form. Even if present, access to such data is often indirect and obscured. This is in particular true in case of delineations of brain structures and their three-dimensional reconstructions like polygonal meshes, labeled volumes or volumetric masks. Hitherto, there is no web service, that readily provides open and programmatic access to 3D models of brain structures for various digital brain atlases.

To address this need we propose the *3D Brain Atlas Reconstructor service* (3dBARs; http://3dbars.org/)—a repository of three-dimensional models of brain structures. The service is an on-line extension of previously developed offline software (Majka et al. 2012b) and utilizes the concept of origin-independent and flexible format for storing delineations or other compatible atlas data: SVG-based Common Atlas Format (CAF). The currently proposed web service plays two roles. First, it serves as a database of publicly available brain atlases of different animals, by different authors, and of varying complexity, provided in a systematized form, the same regardless of the origin of the atlas. Second, it is a repository of three dimensional models of brain structures which means that one can enter the website and browse through the reconstructions, view or fetch them. It also delivers mechanisms for enhancing functionality of other software or websites with the 3dBAR’s features thanks to an application programming interface.

## Implementation

**Fig. 1:**
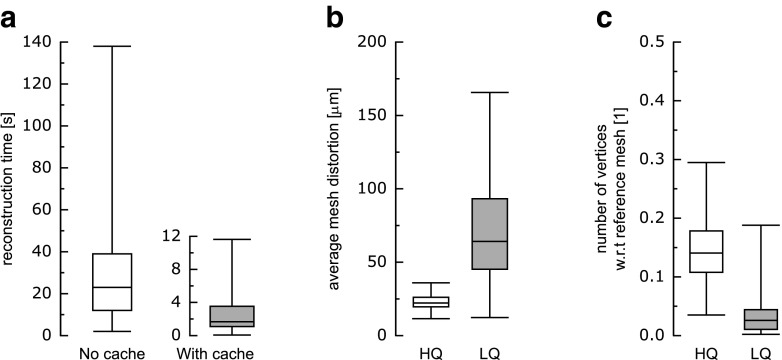
Overview of the 3D Brain Atlas Reconstructor service’s workflow. Boxes represent the main components of the system while arrows indicate interactions between components. Inscriptions next to the arrows describe the nature of the interaction. See the text for a detailed description

The 3D Brain Atlas Reconstructor (http://3dbars.org/) is a client-server web service consisting of several components organized into three layers (Fig. 1). Most of the components were implemented in Python, an open source and free programming language which became popular over last couple of years in scientific applications, among other, in neuroscience context (Davison et al. 2009).

The web server (Fig. 1b) is based on CherryPy (http://www.cherrypy.org/, Hellegouarch (2007))—a lightweight, flexible and configurable HTTP server framework. It is deployed on a Linux machine and uses Psycopg PostgreSQL database adapter (http://initd.org/psycopg/). The web server addresses requests from the native (Fig. 1a) and external (Fig. 1f) client software through the application programming interface (see below), communicates with computational cluster (Fig. 1c) to schedule reconstruction, and with a data repository (Fig. 1e) to manage and serve the content.

The data in the service are stored in two ways: in a PostgreSQL relational database (http://www.postgresql.org/) and in a dedicated file server. The database server holds administrative information needed for user management and authorization data. It also stores entire CAF datasets, which makes it possible to perform queries on the metadata, structures hierarchy as well as on the delineations. Finally, the database server mediates between the reconstruction module (Fig. 1d) and the web server allowing the former to update the status of performed reconstruction and the latter to keep track of the reconstructions available in the system (record the number of structures constituting given reconstruction, available output formats, and similar information). File server holds the actual reconstructions and complementary data like server logs. For the performance purposes the CAF datasets are replicated from the database and stored also on the file server.

The calculations are managed by the TORQUE Resource Manager (http://www.adaptivecomputing.com/products/open-source/torque/). The reconstruction functionality is provided by the previously developed 3D Brain Atlas Reconstructor (Majka et al. 2012b). The software was accommodated by fitting it with the ability to communicate with the database, utilize existing reconstructions and to run under resource manager supervision.

The browser-based web interface (http://3dbars.org/) follows the HTML5 and CSS3 standards. The interface’s functionality is supported by jQuery (http://jquery.com/) framework as well as stand-alone JavaScript scripts. Asynchronous JavaScript and XML (AJAX) queries are used to dynamically communicate with the web server. Three-dimensional visualization is implemented using X3DOM (http://www.x3dom.org/)—a framework for creating web applications using WebGL (http://www.khronos.org/webgl/) technology.

### Available Atlases

At the time of preparing this article we provide 12 atlases publicly available on the internet. Several of them are templates of C57BL/6J inbred strain of house mouse, including consecutive delineations of the Waxholm Space Mouse Brain reference (Johnson et al. 2010), delineations of the symmetrical version of the template (Bowden et al. 2011) and two versions of delineations of the mouse brain from the Allen Institute for Brain Science (Lein et al. 2007). Several other available atlases are based on two dimensional slides provided by the Scalable Brain Atlas project (http://scalablebrainatlas.incf.org/) including a number of templates of macaque monkey’s brain of various complexity (Paxinos et al. 2000; Bowden and Dubach 2003; Kötter and Wanke 2005; Bezgin et al. 2009) and human brain templates (Shattuck et al. 2008; Rohlfing et al. 2010). We later refer to these atlases as the *source atlases* (Fig. 1g). We processed the delineations from the source templates or atlases to the CAF datasets with the offline version of 3D Brain Atlas Reconstructor (Majka et al. 2012b) and uploaded them to the service’s database.

### Pipelines

Computational pipelines are series of Visualization Toolkit (VTK) filters (Schroeder et al. (2006), http://www.vtk.org/) designed to transform the volumetric mask of a given structure into triangular mesh. The set of pipelines is defined separately for each dataset to achieve the best results of the reconstructions. Each atlas is provided with at least three predefined pipelines covering typical applications. The *unprocessed mesh pipeline* provides a mesh derived directly from the volumetric mask by applying marching cubes algorithm (Lorensen and Cline 1987) without any additional processing. This is of use when the user intends to process the mesh further or use it in computations where high reliability of the mesh is required. The *low quality pipeline* produces meshes that are coarse, highly decimated but lightweight and useful for preview purposes. *The high quality pipeline* is a relatively universal way of reconstructing the models and it can be used for visualizations. For some atlases we also provide *smooth mesh pipeline* which produces visually pleasing smooth meshes, especially useful for visualizations, which, however, may lack fidelity as distortions might be introduced while smoothing. For selected datasets mesh distortion was quantified as an average discrepancy between unprocessed meshes and those generated by pipelines that use decimation. Furthermore, the number of vertices constituting each generated mesh was recorded.

### Analysis of the Reconstruction Process

**Fig. 2:**
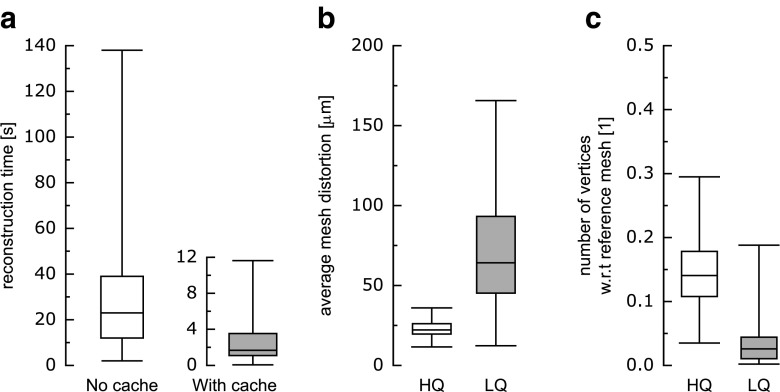
Benchmarking and quality assessment of the service. Box and whisker plots showing order statistics (5 % percentile, lower quartile, median, upper quartile and 95 % percentile) for 197 structures from datasets based on the Waxholm Space mouse brain template. **a** Time elapsed of an initial reconstruction (*No cache*) in comparison with consecutive reconstructions (*With cache*) performed using high quality pipeline. **b** Mesh discrepancy in comparison with unprocessed mesh for high quality pipeline (*HQ*) and low quality pipeline (*LQ*). **c** Comparison of the ratio of vertices from meshes generated with low- and high quality pipeline with respect to the number of vertices in the unprocessed mesh

The reconstruction process is a crucial element of the service’s functionality. The process is triggered by the web server and executed on the computational cluster. As a result, we obtain models, which may consist of one or many structures, and which may be delivered in one or many output formats. The reconstruction request initiated by the server carries (1) a list of structures to be reconstructed, (2) a pipeline that will be used to perform the reconstruction, and (3) the output formats in which the results are to be available. The request is then dispatched by the computational cluster manager to computation nodes so that many reconstructions can be performed simultaneously. On each node a 3dBAR reconstruction module is executed. It checks if a structure to be reconstructed already exists. If that is the case, no actual reconstruction is performed, only a database entry is made that references the existing model with the currently processed request. That allows to avoid model duplications and significantly reduces reconstruction time and resources consumption (Fig. 2a).

If no existing model is found or no precalculated components are available, a reconstruction is performed from the CAF dataset (Majka et al. 2012b). Briefly, CAF slides are stacked into a volumetric mask according to a given volume spacing and passed to the pipeline that produces the polygonal mesh. Volumetric masks are recorded in the data repository so they can be reused by further reconstructions. In the final stage, the reconstructed models are compressed into zip archives to save both storage space and transfer time. The zip file contains the actual model file and a text file with a basic description of the atlas and the licensing information. Additionally, the elapsed time of every reconstruction job is captured for inspection and benchmarking purposes.

### Application Programming Interface

The entire functionality of 3dBARs is available via an HTTP-based application programming interface (API). The core functions of the API include querying the service for available data (both models and atlases), fetching the data, requesting custom reconstructions and monitoring their progress. Auxiliary functions include user registration, signing in, signing out, user’s account and reconstructions management etc. They are necessary for every modern online service but they are not service-specific.

The contents of the repository (reconstructed models, their thumbnails, CAF slides, etc.) can be accessed in multiple ways. For instance, a model can be specified by its unique ID or by a combination of source atlas name and abbreviation of the reconstructed structure. Atlases can be accessed both as labeled volumes and CAF datasets (see the “Results” section for details). Each CAF dataset can be fetched as a zip archive, alternatively, one can access any of its components independently. The full documentation of the API is available trough the Supplementary Materials.

## Results

### Repository of Three Dimensional Reconstructions of Brain Structures

The 3D Brain Atlas Reconstructor service is primarily a repository of three dimensional models of brain structures. The users can choose from several formats of reconstructions according to their requirements. Available formats can be divided into: 1) polygonal meshes 2) volumetric files and 3) images (bitmaps).

The available polygonal mesh formats are Virtual Reality Modeling Language (VRML; http://www.web3d.org/x3d/vrml/), its successor—X3D, ISO standard, XML-based file format (http://www.web3d.org/x3d/specifications/), StereoLithography (STL) format and Visualization ToolKit Poly Data format (http://www.vtk.org/doc/nightly/html/classvtkPolyData.html). This form of reconstruction is commonly used in visualization and can be useful for instance in setting up neural network models for simulations (extending the Java class Region in neuroConstruct (Gleeson et al. 2007) would be one concrete example). The available VRML and X3D formats are able to handle multiple models within a single file while the mechanism for preparing reconstructions in VTK Poly Data and STL supports only a single structure per file.

The second type of reconstructions are volumetric masks of given structures. The masks provide information if a structure is present at a given location within the atlas (mask value of 1) or not (mask value of 0). This type of files if often used in various calculations, e.g., to restrict analysis to a volume of interest (VOI) or to drive label based image registration. The list of available volumetric formats includes: Neuroimaging Informatics Technology Initiative (NIfTI; http://nifti.nimh.nih.gov/) format, Visualization ToolKit Structured Grid format (http://www.vtk.org/doc/nightly/html/classvtkStructuredGrid.html). The volumetric masks can also be accessed as NumPy arrays (http://numpy.scipy.org/).

Both volumetric masks and polygonal meshes maintain coordinates which are derived from the source atlas and provided in RAS orientation convention (X coordinate increases from brain’s left to right, Y increases from posterior to anterior, and Z increases from brain’s inferior to superior).

The last type of reconstructions are bitmaps in PNG format. The images are available in two sizes: $1596\times 1080$ pixels, called a screenshot of the reconstruction, and $276\times 180$ pixels, referred to as a reconstruction thumbnail. Thumbnails are automatically generated in addition to every reconstruction while a screenshot is considered another reconstruction format similarly to the polygonal meshes or volumetric files.

At the moment of preparing the article a total number of over 10 thousand different reconstructions (polygonal meshes and volumetric masks obtained by processing input atlases with various pipelines) of nearly 3000 structures from all the hosted atlases are available. Size of the reconstructions is relatively small as they are based on delineations rather than raster image data and, additionally, stored as zip archives. This allows to both reduce disk space usage and speed up the transfer. All the reconstructions occupy around 4GB of disk space with the file size ranging from tiny 509 bytes up to 170 MB, with the average size of 400 KB.

The efficiency of the reconstruction mechanisms and the quality of the obtained polygonal meshes was evaluated for 197 structures constituting available datasets related to the Waxholm Space mouse brain template (Johnson et al. 2010; Bowden et al. 2011; Hawrylycz et al. 2011) as a representative example in terms of benchmarking (Fig. 2).

The effectiveness of the caching mechanism was assessed by comparing time elapsed on initial and consecutive reconstructions for each of the structures using high quality pipeline (Fig. 2a). It should be noticed that the initial reconstructions, performed with an absence of any precalculated components, took more time to compute (representative 95 % of the reconstructions took up to 138 seconds to complete). In case of the consecutive attempts, the time was reduced by an order of magnitude as cached components were utilized (95 % of the reconstructions took at most 11.6 seconds to compute).

In the next step, the average discrepancy and the ratio of the number of the vertices for models created using low- and high quality pipelines to the number of the vertices of those obtained with unprocessed pipelines were evaluated (Fig. 2b, c). We define the average discrepancy as the average distance between corresponding points of the unprocessed mesh and the evaluated mesh (created with either low- or high quality pipeline). According to this definition, the high quality pipeline produced reconstructions with the average discrepancy at the maximum level of 36 $\mu $m—1.67 times the source volume voxel size (95 % of the reconstructions) while the ratio of the number of vertices with respect to unprocessed mesh extended from 3.5 % up to 28.8 % (for 90 % of the reconstructions). On the other hand, low quality pipeline produces models with much higher distortions as in the representative 95 % of the reconstructions the average discrepancy reached 161 $\mu $m (13.4 times the source volume voxel size). These distortions are a consequence of the high compression of the low quality meshes as the number of vertices in comparison with the unprocessed mesh ranges from 0.2 % to 19.2 % (for the representative 90 % of the reconstructions).

### Available Formats for Atlases

**Fig. 3:**
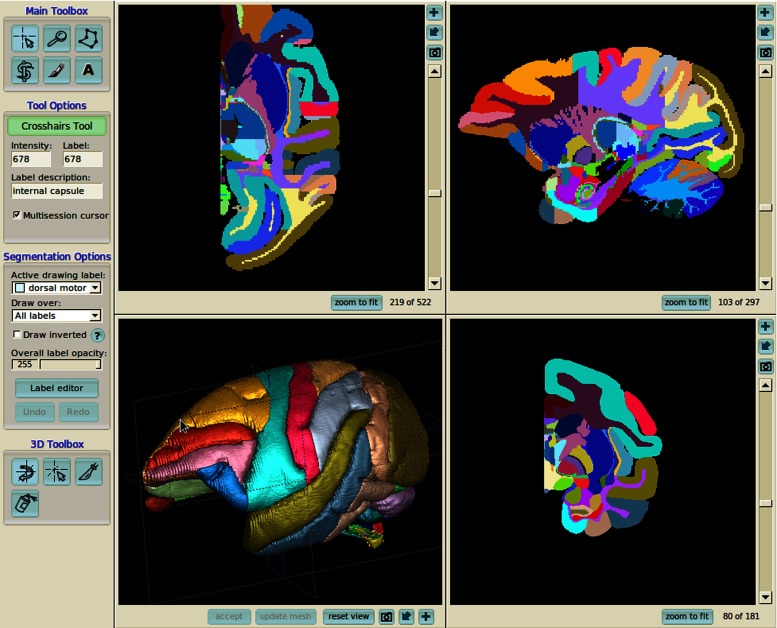
Example of 3dBAR’s data interoperability. Labeled volume of macaque’s brain (Bowden and Dubach 2003) loaded into ITK-SNAP (Yushkevich et al. 2006). Note that the structures’ names, abbreviations and colors are consistent among different forms of the reconstructions throughout the service

Apart from reconstructions representing individual structures or subsets of all structures, two kinds of datasets carry all delineations available within a given atlas. The first one is a NIfTI file with labeled volume based on the atlas. In addition to the volume, a look-up table is provided which contains a mapping from the label index to the corresponding structure’s name, color etc. This allows the labeled volume to be used with a wide range of neuroimaging software like 3d Slicer (http://www.slicer.org/; Pieper et al. 2004), ITK-SNAP (Yushkevich et al. 2006) or similar (Fig. 3). Another type of dataset containing all the data constituting the atlas is the Common Atlas Format (CAF) dataset from which all the reconstructions originate (see the “Implementation” section). Because of that, names, abbreviations, colors, etc. are consistent among the different formats. All the types of reconstructions and datasets including those containing the whole atlas are available for every atlas regardless of its source form (either 2D or 3D).

### Web Interface

Although the 3D Brain Atlas Reconstructor service provides programmatic ways of accessing the data, a typical user would probably start exploring the service from a browser-based interface which we provide (http://3dbars.org/). Here we highlight only the key elements as the full documentation of the interface, including tutorials and screencasts illustrating every feature in detail, is provided in the Supplementary Materials.

### Collection of Brain Atlases of Different Species of Animals

One of the major roles of the 3dBARs is to host a collection of brain atlases of various species of animals. For every atlas a range of metadata is provided including information like: sex, age, body weight, species, strain, etc. of animals used to create the atlas. The amount of provided metadata varies between atlases as some of them contain richer description than others. Moreover, for every atlas we provide a reference to the exact file(s) that have been used as a basis for generation of the 3D reconstructions. Whenever possible, licensing information for the atlases is provided. In some cases the information is displayed in simplified form suggested by Creative Commons (http://creativecommons.org/), in other—by pointing to the author’s or publisher’s website with citation or data sharing policy for the given atlas.

### Interactive Browser of Three Dimensional Models of Brain Structures

An integral part of the 3dBARs is an interactive browser of three dimensional models of brain structures. The interface allows the user to visualize lightweight versions of 3D models of brain structures available in hosted atlases in the browser window. The interface is available through a web browser and is intended to work with any modern web browser which supports WebGL technology (http://www.khronos.org/webgl/). At the time of writing we found that Google Chrome provides the most reliable support for WebGL, see also the “Discussion and Summary”. All the results presented here were tested with Google Chrome version 23, both in Linux and Windows operating systems. As opposed to, for example, Java applications, there is no need for installing or downloading any additional software to use the interface.

**Fig. 4:**
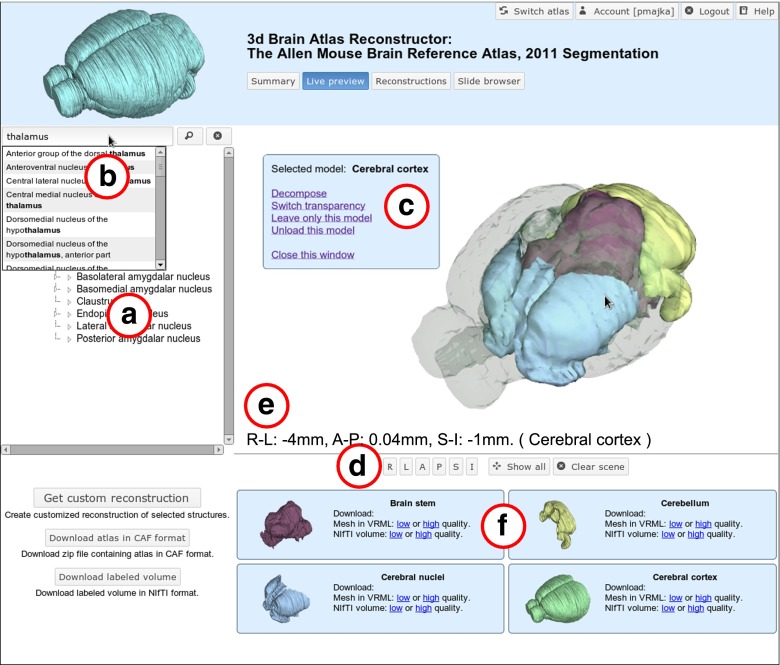
Example scene from the live preview interface with selected models of structures from the Allen Mouse Brain Reference Atlas (http://mouse.brain-map.org/): brain stem (*brown*), cerebellum (*yellow*), cerebral nuclei (*blue*) and cerebral cortex (*green, transparent*). See text for description of highlighted regions (**a–f**)

Figure 4 shows an example live preview window with a set of reconstructions from the Allen Mouse Brain Reference Atlas (http://mouse.brain-map.org/). The user has a wide range of interaction options available. He or she can look for and display structures from the hierarchy tree (Fig. 4a), search for a particular structure from the list of structures using auto complete function (Fig. 4b), zoom the scene in and out, rotate the models, apply transparency, load multiple structures based on the hierarchy (Fig. 4c), see the structures from different angles, apply predefined viewpoints (right, left, anterior, posterior, superior, inferior, perspective, etc.; Fig. 4d), read out the coordinates of the location pointed by the mouse cursor (Fig. 4e). All models currently loaded are listed below the preview window as tiles summarizing precalculated reconstructions which are ready for immediate download (Fig. 4f). The tiles include the visualization (thumbnail) and download links for polygonal mesh and volumetric mask in both *high* and *low* reconstruction preset.

### Reconstruction Browser

Apart from the default, precalculated set of reconstructions, we provide the *reconstruction browser*, an interface that allows to search for specific reconstructions by their name, resolution, computational pipeline, available file format etc. The results come from the whole repository including the precalculated structures, additional reconstructions generated by the administrators as well as reconstructions generated by users using the *Custom Reconstruction Wizard* (see below). The results of the query can be downloaded selectively, alternatively the user can access a set of links to all the results.

### Custom Reconstruction Wizard

Although precalculated reconstructions are available in two quality presets and two formats, it commonly happens that users require reconstructions performed in a different way or in a format in which the precalculated reconstructions are not available. In such case users may access the Custom Reconstruction Wizard. The wizard is a part of the browser-based interface and allows to submit a request for reconstruction according to one’s requirements or specification. The reconstruction is performed entirely on the server side thus it does not require installation of additional software and does not utilize resources of the client’s computer.

The wizard can be accessed in two ways: from live preview window or through the reconstruction browser. In the first case, all the structures currently displayed in the preview window are automatically transferred to the wizard as a set of structures to be reconstructed. In the latter case, the structure for which the query was performed is transferred to the wizard. Since the mechanism requires the use of our internal computer cluster, it can only be accessed by registered users. Default limit of the reconstruction jobs for a single user is twenty per day. It may be changed to an arbitrary value or even withdrawn for a particular user upon his or her request. Reconstructions created using the wizard can be managed (tracked, reviewed, deleted, etc.) through the user account.

**Fig. 5:**
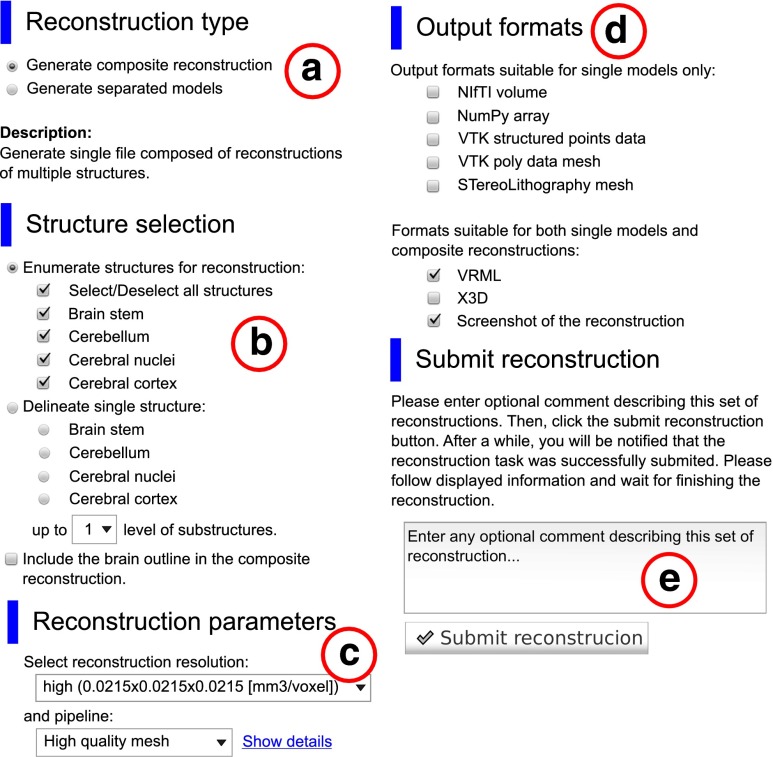
Consecutive tabs constituting the Custom Reconstruction Wizard. The tabs were extracted from the interface and put together into a single figure to maintain legibility. Structures from Fig. 4 are loaded into the wizard. The presented settings lead to a reconstruction with multiple structures within a single file (*“composite reconstruction”*, panel **a**) of brain stem, cerebellum, cerebral nuclei and cerebral cortex (panel **b**) that is going to be conducted with the “high resolution pipeline” (panel **c**). The output reconstruction will be available as VRML and PNG file (panel **d**, *at the bottom*). Panel **e** shows **a** possibility to add optional extra information

When using the wizard, the user may customize the following features and parameters of the reconstruction. Primarily, the reconstruction can be composed either from a single structure or an arbitrary set of structures belonging to a given atlas (Fig. 5a, b). The user can choose the computational pipeline from a list of available pipelines and the underlying source volume spacing (Fig. 5c). Apart from the verbal description, the explicit scheme of the pipeline may be displayed. In addition, reconstructions may be generated in all mentioned file formats depending on user’s requirements (Fig. 5d). Each reconstruction request can also be annotated with a comment describing, e.g., the purpose of the reconstruction or the structures included (Fig. 5e). All reconstructions conducted through the wizard are automatically publicly available.

### Slide Browser

As a complementary feature we also provide a simple slide browser. It provides the user with access to the set of two dimensional slides that constitute the basis for three-dimensional reconstructions. Each slide has a coordinate of its plane displayed. The user can navigate to the next, previous, or to any other slide from the atlas and hide or show the labels with the structure’s abbreviations. He or she can also download the currently viewed image (in SVG-based CAF slide format) for further processing or for use along with experimental data, etc.

### User Account Panel

Registered users can access user panel which gives access to their account data. The panel allows the user to track his activity, review the reconstructions conducted, read out the account usage information, check the number of reconstructions available for a given day, etc. The user can edit his personal data (change name, password, etc.) or terminate the account.

## Discussion and Summary

### Overall Functionality

We provide a complete and operational online service with documentation and tutorials. We implemented a comprehensive set of features related to distribution and processing of brain atlases and 3D reconstructions of brain structures. As an online system, 3dBARs can be accessed easily without necessity to install any software or plugins on the local computer. The service offers storing and managing of brain atlases of different origin, complexity and form (e.g., based on 2D or 3D delineations, derived from publications or web sites, etc.). The service gathers metadata from the source atlases and plays a role of an atlas database. The service also provides references to the source data used to generate the 3D reconstructions making the whole process fully reproducible. Licensing information helps the user to determine the allowed usage of the atlas. The user can easily find each source atlas and its description or contact the author of the particular atlas himself.

The main purpose of the service is to provide open and programmatic access to 3D models of brain structures from various brain atlases. 3dBARs realizes this function by distributing the reconstructions in a unified form independent from the form of the atlas source data. The service provides a number of data formats in which the delineations can be accessed. This includes polygonal mesh formats, volumetric file formats and vector graphics slides. Moreover, all the data are accessible interactively using the browser-based interface as well as programmatically—with a dedicated application programming interface. The service is a dynamic repository as it provides an on-demand mechanism for generating users’ own reconstructions which are distributed similarly as the native reconstructions. As the reconstructions are generated entirely on the server side and are publicly available, they can be shared among other users, e.g. collaborators, very easily, by providing the link to the reconstruction.

The service is a natural extension of the previously developed 3D Brain Atlas Reconstructor software to the internet. For that reason both mechanisms are fully compatible: The CAF datasets available via the service can be downloaded and used with the offline version of the 3d Brain Atlas Reconstructor.

### Architecture

The architecture of the system is not limited to the data modality we use, which are delineations. On the contrary, the architecture, the overall organization (presentation layer, server layer, computation layer) and the data flow may be used for other web services of similar philosophy, especially for other atlasing services performing on-demand calculations. Setting up two servers, one responsible for serving the data and the other concerned only with computations allowed us to separate handling the clients’ requests and performing computations. This makes the system more stable and resistant to failures. For example, in case of a breakdown of the computation cluster, the web server can continue and the service loses only a part of its functionality. The drawback of this solution is lower performance in short or interactive calculations.

### Limitations of the Live Preview Feature and the WebGL Technology

The main source of the limitations is the current state of the available technology. This is especially visible in the *live preview* interface. Our 3D viewer is based on relatively new WebGL technology introduced with the HTML5 standard. Although a part of the standard, currently only some of the major browsers support the technology. In our experience, Google Chrome provides the most stable support for WebGL across different platforms and versions. Mozilla Firefox supports WebGL, unfortunately the quality of implementation varies across different versions and systems. Nevertheless, we decided to use WebGL as it is one of the most promising technologies evolving on the Internet and we believe that with the development of the browsers the handling of WebGL will significantly improve and stabilize.

The same remark applies to the WebGL frameworks in general. Although a number of them is available (see http://www.khronos.org/webgl/wiki/User_Contributions#Frameworks for a comprehensive list of WebGL frameworks), according to our survey done at the moment of creating the service, no framework supported all the features we were planning to use. Some of them lacked convenient functions for even basic operations, like setting particular viewpoint or editing properties of the polygonal mesh, such as color or transparency. We decided to use X3DOM framework, however, since we started, many new WebGL frameworks have emerged, most of them focused on entertainment applications such as game development. One of the most promising endeavors, particularly suitable for scientific purposes is The X Toolkit (XTK; https://github.com/xtk/Xi/). It attempts to mimic the VisualizationToolkit library syntax in functions’ and methods’ names and appears to be geared more towards scientific visualization than the other frameworks. If we started development of the 3dBARs now, the XTK would probably be our framework of choice.

### Limitations of the Reconstruction System

One may find a few limitations in the Custom Reconstruction Wizard in comparison to the offline version of the 3D Brain Atlas Reconstructor. Using the former one can choose a particular pipeline from a set of available pipelines realizing some generic wokflows (e.g. smooth, raw, detailed), but the pipelines themselves cannot be altered. The viewing angle and background colors of the screenshots are fixed and the transparency may be applied only to the root brain structure. These limitations were imposed deliberately as freeing all the options would considerably increase the number of possible reconstruction options which would require immediate feedback from the user as he or she would like to frequently review the results of the changes. All the omitted functionalities are commonly available in almost any 3D visualization software such as 3d Slicer (http://www.slicer.org/; Pieper et al. 2004), Kitware ParaView (http://www.paraview.org/), BioImage Suite, (http://bioimagesuite.yale.edu/) or in the offline version of 3D Brain Atlas Reconstructor (http://3dbar.org/).

### Interoperability with Other Neuroinformatics Software

A typical atlasing repository hosts numerous kinds of data from a single institution along with appropriate interfaces for accessing their data such as gene expression, connectivity, high resolution histology, MRI data, etc. Instead of duplicating these efforts by providing just another interface, the philosophy of 3dBARs is different. The role of the 3D Brain Atlas Reconstructor service could be expressed as *a brick in the wall of neuroinformatics software* as it draws the data from existing atlasing repositories at the same time providing them with three-dimensional reconstructions or delineations which can be used for visualization or computations. We endorse such an approach as most likely no single software or web service will provide functionality covering every type of usage of atlas data. Thus it is important to provide specialized services that can relatively easily equip a particular dataset, website or software with specific functionality. This is the Unix philosophy in the world of web services.

A modern data repository should provide a convenient interface for data exchange and allow integration of its services with other systems or software. Interoperability and data sharing tasks are realized by 3dBARs’ Application Programming Interface consisting of a set of HTTP queries. They provide developers with programmatic access to all the resources and service’s features including: browsing and querying for the reconstructions, downloading the reconstructions in various data formats, submitting the custom reconstructions and tracking their status as well as managing the authentication and user accounts. Any website or software can access and use 3dBARs’ data, for instance, by cross-linking the reconstructions or visualizations of the structures.

Currently, 3D Brain Atlas Reconstructor service enhances functionality of three other web services. ScalableBrainAtlas (http://scalablebrainatlas.incf.org/) established a plug-in that connects to our service and requests visualization of the structures and links to reconstructions. Similar functionality is utilized by NeuroLex, the Neuroscience Lexicon wiki (http://neurolex.org/). 3dBARs supplies also some INCF Digital Atlasing Infrastructure Task Force WaxML requests (http://code.google.com/p/incf-dai/wiki/WaxMLi/) with links to 3D reconstructions. It is an interesting example as it shows how the services can be organized in a cascading way where one service can carry the results from a number of other services.

### General Neuroscience Context

Neuroanatomical atlases are basic tools of neuroscience. They are used by neuroscientists to find the way through the brain during brain slice staining, stereotaxic surgeries and as reference frames for data registration. 3D Brain Atlas Reconstructor service provides a convenient way to access atlas data for several animals in both 2D and 3D formats. Since the service is an open resource it can play a major role in education. In research, the added value of 3D context can be seen in all studies requiring the knowledge of geometrical relations between different parts of the brain. For instance, in design of slices preserving certain connections, in modeling studies of extracellular potential, in volumetric registration of gene expression data, etc. The data located in 3dBARs are available in multiple formats which lowers the barrier for integration of the data with one’s own.

Coregistration of data of different origins is a major challenge today. One of the problems is the lack of standard transformations between different atlases,especially those of small laboratory animals, another is a lack of efficient software which would allow to easily register various experimental data in a given atlas space. Although the 3D Brain Atlas Reconstructor service does not provide dedicated coregistration tools, it will facilitate coregistration by providing different atlases in the same, compatible format and, by this, eliminating a lot of preprocessing steps which would be necessary otherwise. A good example would be a coregistration of some cytoarchitectonic brain atlas based on delineated stained slices with some MRI template. We believe that the open data and proposed interfaces offering a convenient access to 2D and 3D atlases are important contribution in establishing practical coregistration workflows.

### Future Developments

Currently planned future developments of 3dBARs include two directions. First of all, we wish to increase the number of datasets available in the service. In this respect we collaborate with colleagues working on different species towards this goal. New datasets will be announced on 3dBARs website as they become available. Currently, it is not possible for a user to add a new atlas dataset himself. We decided that such events will be too rare to justify developing a dedicated user-centered mechanisms and provide external quality control. At the same time we have sufficient capacity to handle it on administrators side and we encourage parties interested in providing their own data to contact us.

An extension which we plan in the context of currently developed atlas of *Monodelphis* opossum’s brain (Chlodzinska et al. 2012; Majka et al. 2012a) is connecting 3dBARs with the virtual microscopy services. We envision a possibility of connecting a 3D model view or delineations directly with the corresponding location in the underlying high-resolution histology data according to the appropriate spatial coordinates. The details will be reported elsewhere.

## Information Sharing Statement

The 3D Brain Atlas Reconstructor web service can be accessed at http://3dbars.org/. All the data and examples presented in this article are available trough the website. Supplementary materials (available at http://www.3dbar.org/wiki/barServiceSupplement/) contain information on reproducing results, documentation of the Application Programming Interface, detailed tutorials and screencasts on all the features of the service.

## Electronic supplementary material

Below is the link to the electronic supplementary material.

(PDF 1.22 MB)
