# Supplementary material for: 3D Brain Atlas Reconstructor Service—Online Repository of Three-Dimensional Models of Brain Structures
Source: Neuroinformatics. 2013 Aug 14;11(4):507–18. doi: 10.1007/s12021-013-9199-9 (PMC3824210; doi:10.1007/s12021-013-9199-9)
Supplement: Supplementary file 1 — (PDF 1.22 MB) [file 12021_2013_9199_MOESM1_ESM.pdf]

Supplementary material

Web page http://www.3dbar.org/wiki/barServiceSupplement contains the most recent versions of supplementary materials for the article:

3D Brain Atlas Reconstructor service - online repository of three-dimensional models of brain structures

Piotr Majka(*), Jakub M. Kowalski, Natalia Chlodzinska, Daniel K. Wójcik

ABSTRACT

Brain atlases are important tools of neuroscience. Traditionally prepared in paper book format, more and more commonly they take digital form which extends their utility. To simplify work with different atlases, to lay the ground for developing universal tools which could abstract from the origin of the atlas, efforts are being made to provide common interfaces to these atlases. 3D Brain Atlas Reconstructor service (3dBARs) described here is a repository of digital representations of different brain atlases in CAF format which we recently proposed and a repository of 3D models of brain structures. A graphical front-end is provided for creating and viewing the reconstructed models as well as the underlying 2D atlas data. An application programming interface (API) facilitates programmatic access to the service contents from other websites. From a typical user's point of view, 3dBARs offers an accessible way to mine publicly available atlasing data with a convenient browser based interface, without the need to install extra software. For a developer of services related to brain atlases, 3dBARs supplies mechanisms for enhancing functionality of other software. The policy of the service is to accept new datasets as delivered by interested parties and we work with the researchers who obtain original data to make them available to the neuroscience community at large. The functionality offered by the 3dBARs situates it at the core of present and future general atlasing services tying it strongly to the global atlasing neuroinformatics infrastructure.

Following supplementary materials are available:

1. Documentation of the Application Programming Interface (API)
2. Tutorials:
   - How to access the Custom Reconstruction Wizard?
   - How to use labeled volumes available at 3dBAR on-line service website?
3. Screencasts:
   - A whole playlist:
   - Selecting an atlas,
   - Displaying atlas details,
   - Switching the atlas,
   - Browsing atlas slides,
   - 3D model preview,
   - Browsing reconstructed models,
   - Requesting a custom reconstruction,
   - Using the user panel,
   - Other features of 3D model preview,
   - Requesting a composite custom reconstruction.
4. Troubleshooting

Reproducing results from the article		
Figure 2	Figure 3	Figure 4
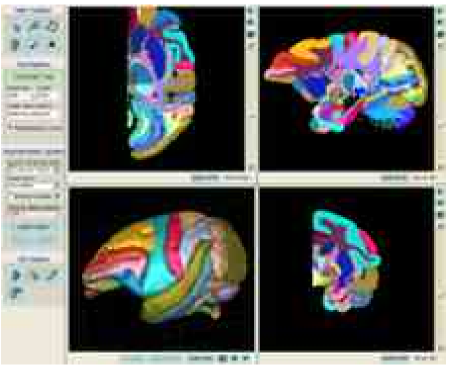	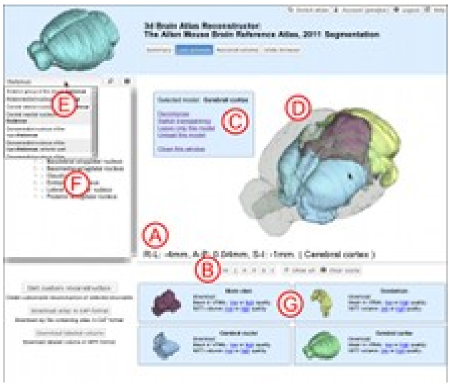	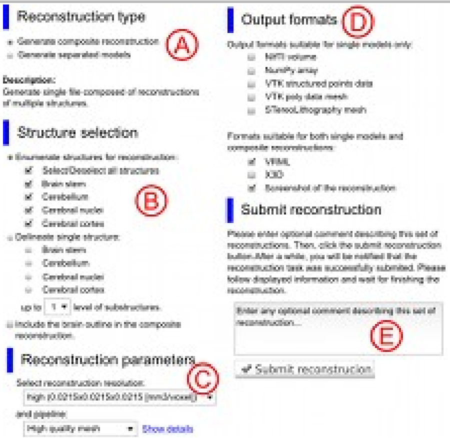
Reproducing Figure 2: Labeled volume of Macaque's brain (Bowden et. al. 2003) loaded into ITK-SNAP (Yushkevich et. al. (2006).	Reproducing Figure 3: Models of structures from the Allen Mouse Brain Reference Atlas (http://mouse.brain-map.org/):<br>• brain stem,<br>• cerebellum,<br>• cerebral nuclei,<br>• cerebral cortex.	The output from the Custom Reconstruction Wizard according to settings from Figure 4:<br>• VRML scene,<br>• PNG image,<br>• reconstruction job tracking page.

Attachments

INNOVATIVE ECONOMY
NATIONAL COHESION STRATEGY
EUROPEAN UNION
EUROPEAN REGIONAL DEVELOPMENT FUND

3d Brain Atlas Reconstructor on-line service

1. Backend services
   1. getAvailableDatasets
   2. getCaf
   3. getCafInfo
   4. getCafSlide
   5. getVolume
   6. getReconstruction
   7. getPreviewReconstruction
   8. getThumbnail
   9. queryReconstruction
   10. getReconstructionByKey
   11. getThumbnailByKey
2. Frontend services
   1. getPreview
   2. getCafInfoPage
   3. queryJob

Backend services

getAvailableDatasets

Return AJAX data describing CAF datasets (atlases) available to the user in the service. See details.

Example

- http://service.3dbar.org/getAvailableDatasets

getCaf

Return zip archive containing requested CAF dataset. See details

Example

- http://www.3dbar.org:8080/getCaf?cafDatasetName=whs_0.6.1

getCafInfo

Return the index file of selected CAF dataset. Index file contains the list of structures available for reconstruction, their hierarchy and the list of slides within the given dataset. See details.

Examples

- http://service.3dbar.org/getCafInfo?cafDatasetName=whs_0.51
- http://service.3dbar.org/getCafInfo?cafDatasetName=whs_0.51;listSlides=False;listStructures=False

getCafSlide

Return an SVG image with CAF slide filtered using provided settings. See details.

Examples

- http://service.3dbar.org/getCafSlide?cafDatasetName=whs_0.51;slideNumber=460
- http://service.3dbar.org/getCafSlide?cafDatasetName=whs_0.51;slideNumber=460;showMask=True;showLabels=False
- http://service.3dbar.org/getCafSlide?cafDatasetName=whs_0.51;slideNumber=460;showLabels=False
- http://service.3dbar.org/getCafSlide?cafDatasetName=sba_DB08;slideNumber=84;structureList=Cx
- http://service.3dbar.org/getCafSlide?cafDatasetName=sba_DB08;slideNumber=84;structureList=Cx,Th

getVolume

Return zipped labeled NifTi? volume containing neuroanatomical information from the requested CAF dataset. See details

Example

- http://www.3dbar.org:8080/getVolume?cafDatasetName=whs_0.6.1

getReconstruction

Return a model of a given structure from selected dataset as a zip file containing the reconstruction and a short description. See details.

Examples

- http://service.3dbar.org/getReconstruction?cafDatasetName=whs_0.51;structureName=thalamus
- http://service.3dbar.org/getReconstruction?cafDatasetName=whs_0.51;structureName=thalamus;qualityPreset=high;outputFormat=vrml

getPreviewReconstruction

Return a lightweight model of a given structure from selected dataset as an x3d mesh. See details.

Examples

- http://service.3dbar.org/getPreviewReconstruction?cafDatasetName=whs_0.51;structureName=thalamus
- http://service.3dbar.org/getPreviewReconstruction?cafDatasetName=sba_DB08;structureName=Br

getThumbnail

Return a thumbnail of the reconstruction of a given structure available via `getReconstruction` in png format. See details.

Examples

- http://service.3dbar.org/getThumbnail?cafDatasetName=whs_0.51;structureName=corpus-callosum
- http://service.3dbar.org/getThumbnail?cafDatasetName=whs_0.51;structureName=thalamus
- http://service.3dbar.org/getThumbnail?cafDatasetName=whs_0.51;structureName=CNS
- http://service.3dbar.org/getThumbnail?cafDatasetName=sba_DB08;structureName=FL
- http://service.3dbar.org/getThumbnail?cafDatasetName=sba_DB08;structureName=LV
- http://service.3dbar.org/getThumbnail?cafDatasetName=sba_PHT00;structureName=Brain

queryReconstruction

Return AJAX data describing reconstructions in the service matching the criteria. See details.

Examples

- http://service.3dbar.org/queryReconstruction?cafDatasetName=sba_DB08;structureList=Br;outputFormats=exportToVRML
- http://service.3dbar.org/queryReconstruction?cafDatasetName=whs_0.6.1;structureList=Brain;outputFormats=exportToVRML,exportToX3d;pipeline=whs_0.6.1-LQ;resolution=0.043,0.043
- http://service.3dbar.org/queryReconstruction?cafDatasetName=sba_DB08;structureList=Amg;switches=brainoutline;outputFormats=exportToVRML,exportToX3d,exportScreenshot

getReconstructionByKey

Return reconstruction (stored in the service) of a given service identifier. The identifier can be obtained using 'queryReconstruction' service. See details.

Example

- http://service.3dbar.org/getReconstructionByKey?id=26721

getThumbnailByKey

Return a thumbnail of reconstruction (stored in the service) of a given service identifier in png format. See details.

Example

- http://service.3dbar.org/getThumbnailByKey?id=12064

Frontend services

getPreview

Display a window allowing to manipulate (rotate, zoom, etc.) lightweight version of the reconstuciton. Note that the web browser has to support WebGL in order to use this feature. See details.

Examples

- http://service.3dbar.org/getPreview?cafDatasetName=whs_0.51&structureName=CNS
- http://service.3dbar.org/getPreview?cafDatasetName=whs_0.5&structureName=Hc
- http://service.3dbar.org/getPreview?cafDatasetName=sba_DB08&structureName=FBr

getCafInfoPage

Display a window containing basic information about CAF dataset. See details.

Examples

- http://service.3dbar.org/getCafInfoPage?cafDatasetName=sba_DB08

queryJob

Display a window containing information about a reconstruction job of requested identifier - and about other reconstruction jobs requested for the same CAF dataset. See details.

Examples

- http://service.3dbar.org/queryJob?id=22

INNOVATIVE ECONOMY
NATIONAL COHESION STRATEGY
EUROPEAN UNION
EUROPEAN REGIONAL DEVELOPMENT FUND

3d Brain Atlas Reconstructor on-line service

getAvailableDatasets

Return an AJAX data describing CAF datasets (atlases) available to the user in the service.

syntax

```
getAvailableDatasets
```

AJAX data type

```
{
  String: [String,
           String,
           String,
           String,
           {
             String: Boolean,
             ...
           },
           String],
  ...
}
```

AJAX data description

The data is provided in format:

```
{
  <id>: [<name>,
         <root>,
         <description>,
         <date>,
         {
           "cafslides": <cafslides>,
           "reconstructions": <reconstructions>,
           "thumbnails": <thumbnails>,
           "customreconstruction": <customreconstruction>,
           "hidden": <hidden>,
           "internal": <internal>,
           "enabled": true
         },
         <unit>],
  ...
}
```

where:

<id>
: is the atlas identifier in the service,

<name>
: is the atlas name,

<root>
: is the name of the root element in the atlas structures hierarchy tree,

<description>
is the atlas description,

<date>
is the CAF dataset compilation date,

<cafslides>
indicates if CAF slides are available,

<reconstructions>
indicates if reconstructions of structures in the atlas are available,

<thumbnails>
indicates if thumbnail images of structures in the atlas are available,

<customreconstruction>
indicates if it is possible to submit custom reconstruction of structures in the atlas,

<hidden>
indicates if the dataset is invisible in the front-end interface,

<internal>
indicates if access to the dataset is restricted to limited group of users,

<unit>
is the spacial reference system unit used in the dataset.

Example

- http://service.3dbar.org/getAvailableDatasets

INNOVATIVE ECONOMY
NATIONAL COHESION STRATEGY
EUROPEAN UNION
EUROPEAN REGIONAL DEVELOPMENT FUND

3d Brain Atlas Reconstructor on-line service

getCaf

Return zip archive containing requested CAF dataset.

syntax

```
getCaf?cafDatasetName=<name>
```

Required arguments

cafDatasetName
The service identifier of the selected CAF dataset.

Example

- http://www.3dbar.org:8080/getCaf?cafDatasetName=whs_0.6.1

INNOVATIVE ECONOMY
NATIONAL COHESION STRATEGY
EUROPEAN UNION
EUROPEAN REGIONAL DEVELOPMENT FUND

3d Brain Atlas Reconstructor on-line service

getCafInfo

Return the index file of selected CAF dataset. Index file contains list of structures available for reconstruction, their hierarchy and list of slides within given dataset.

syntax

```
getCafInfo?cafDatasetName=<name>
          [;listStructures=(True|False)]
          [;listHierarchy=(True|False)]
          [;listSlides=(True|False)]
```

Required arguments

cafDatasetName
The service identifier of the selected CAF dataset.

Optional arguments

All optional arguments may be set to True or False with True as default.

listStructures
Indicates if information about structures has to be included in the CAF index file.

listHierarchy
Indicates if information about structures hierarchy has to be included in the CAF index file.

listSlides
Indicates if information about slides has to be included.

Examples

- http://service.3dbar.org/getCafInfo?cafDatasetName=whs_0.51
- http://service.3dbar.org/getCafInfo?cafDatasetName=whs_0.51;listSlides=False;listStructures=False

3d Brain Atlas Reconstructor on-line service

getCafSlide

Return a SVG image with CAF slide filtered by provided settings.

syntax

```
getCafSlide?cafDatasetName=<dataset name>
            ;slideNumber=<slide number>
           [;structureList=[<structure #1>[,<structure #2>[...]]]]
           [;showLabels=(True|False)]
           [;showMask=(True|False)]
```

Required arguments

cafDatasetName
: The service identifier of the selected CAF dataset.

slideNumber
: Number of the CAF slide according to `slideindex` section of CAF index file.

Optional arguments

structureList
: Comma-separated List of structures. If provided only structures from the list are included in returned CAF slide. If not specified, the returned CAF slide contains all structures.

showLabels
: Indicates if labels are to be included in the generated slide. Can be set to `True` or `False`. `True` by default.

showMask
: If True, provides black & white mask of the slide and forces labels to be removed. `False` by default.

Examples

- http://service.3dbar.org/getCafSlide?cafDatasetName=whs_0.51;slideNumber=460
- http://service.3dbar.org/getCafSlide?cafDatasetName=whs_0.51;slideNumber=460;showMask=True;showLabels=False
- http://service.3dbar.org/getCafSlide?cafDatasetName=whs_0.51;slideNumber=460;showLabels=False

INNOVATIVE ECONOMY
NATIONAL COHESION STRATEGY

EUROPEAN UNION
EUROPEAN REGIONAL
DEVELOPMENT FUND

3d Brain Atlas Reconstructor on-line service

getVolume

Return zipped labeled NifTi? volume containing neuroanatomical information from the requested CAF dataset.

syntax

```
getVolume?cafDatasetName=<name>
```

Required arguments

cafDatasetName
The service identifier of the selected CAF dataset.

Example

- http://www.3dbar.org:8080/getVolume?cafDatasetName=whs_0.6.1

INNOVATIVE ECONOMY
NATIONAL COHESION STRATEGY
EUROPEAN UNION
EUROPEAN REGIONAL DEVELOPMENT FUND

3d Brain Atlas Reconstructor on-line service

getReconstruction

Return a model of a given structure from selected dataset as a zip file containing the reconstruction and short description.

syntax

```
getReconstruction?cafDatasetName=<dataset name>
                  ;structureName=<structure name>
                 [;qualityPreset=(low|high)]
                 [;outputFormat=(vrml|niftii)]
```

Required arguments

cafDatasetName
The service identifier of the selected CAF dataset.

structureName
Name of structure from hierarchy tree. In practice nearly all names should be fine. Formally name has to point to a root node of a subtree containing at least one node with representation among paths in CAF slides.

Optional arguments

qualityPreset
Sets predefined quality preset. Possible values are 'low' and 'high', the default value is 'low'.

outputFormat
Defines format of the reconstruction. Possible values are 'vrml' and 'niftii', the default value is 'vrml'.

Examples

- http://service.3dbar.org/getReconstruction?cafDatasetName=whs_0.51;structureName=thalamus
- http://service.3dbar.org/getReconstruction?cafDatasetName=whs_0.51;structureName=thalamus;qualityPreset=high;outputFormat=vrml

INNOVATIVE ECONOMY
NATIONAL COHESION STRATEGY
EUROPEAN UNION
EUROPEAN REGIONAL DEVELOPMENT FUND

3d Brain Atlas Reconstructor on-line service

getPreviewReconstruction

Return a lightweight model of a given structure from selected dataset as an x3d mesh.

syntax

```
getPreviewReconstruction?cafDatasetName=<dataset name>
                             ;structureName=<structure name>
```

Required arguments

cafDatasetName
The service identifier of the selected CAF dataset.

structureName
Name of structure from hierarchy tree. In practice nearly all names should be fine. Formally name has to point to a root node of a subtree containing at least one node with representation among paths in CAF slides.

Examples

- http://service.3dbar.org/getPreviewReconstruction?cafDatasetName=whs_0.51;structureName=thalamus

INNOVATIVE ECONOMY
NATIONAL COHESION STRATEGY
EUROPEAN UNION
EUROPEAN REGIONAL DEVELOPMENT FUND

3d Brain Atlas Reconstructor on-line service

getThumbnail

Return a thumbnail of the reconstruction of a given structure available via getReconstruction in png format.

syntax

```
getThumbnail?cafDatasetName=<dataset name>
             ;structureName=<structure name>
```

Required arguments

cafDatasetName
The service identifier of the selected CAF dataset.

structureName
Name of structure from hierarchy tree. In practice nearly all names should be fine. Formally name has to point to a root node of a subtree containing at least one node with representation among paths in CAF slides.

Examples

- http://service.3dbar.org/getThumbnail?cafDatasetName=whs_0.51;structureName=corpus-callosum
- http://service.3dbar.org/getThumbnail?cafDatasetName=whs_0.51;structureName=thalamus
- http://service.3dbar.org/getThumbnail?cafDatasetName=whs_0.51;structureName=CNS
- http://service.3dbar.org/getThumbnail?cafDatasetName=sba_DB08;structureName=FL
- http://service.3dbar.org/getThumbnail?cafDatasetName=sba_DB08;structureName=LV
- http://service.3dbar.org/getThumbnail?cafDatasetName=sba_PHT00;structureName=Brain

INNOVATIVE ECONOMY
NATIONAL COHESION STRATEGY
EUROPEAN UNION
EUROPEAN REGIONAL DEVELOPMENT FUND

3d Brain Atlas Reconstructor on-line service

queryReconstruction

Return AJAX data describing reconstructions in the service matching the criteria.

syntax

```
queryReconstruction?cafDatasetName=<dataset identifier>
                   ;structureList=<structure name>
                   ;outputFormats=<output format list>
                  [;resolution=<resolution>]
                  [;pipeline=<pipeline name>]
                  [;switches=<switches list>]
```

Required arguments

cafDatasetName
: The service identifier of the selected CAF dataset.

structureName
: Name of structure from hierarchy tree. In practice nearly all names should be fine. Formally name has to point to a root node of a subtree containing at least one node with representation among paths in CAF slides.

outputFormats
: Nonempty coma-separated list of reconstruction file format descriptors. Allowed format descriptors are:

- 'exportToVRML': VRML files,
- 'exportToX3d': X3D files,
- 'exportToPOVRay': POV-Ray files,
- 'exportToNiftii': NIfTI files
- 'exportToVTKPolydata': vtk polydata files,
- 'exportToNumpy': NumPy? array files,
- 'exportToVolume': vtk structured grid files,
- 'exportScreenshot': PNG images,
- 'exportThumbnail': PNG thumbnails.

Optional arguments

resolution
: Coronal and anterior-posterior voxel size separated with a coma.

pipeline
: Service identifier of the pipeline the reconstructions has been generated with.

switches
: Nonempty coma-separated list of switches. Allowed switches are:

- 'brainoutline': match only structures containing the outline of hierarchy tree root structure; forces 'composite' switch,
- 'composite': only scene format descriptors ('exportToVRML', 'exportToX3d', 'exportToPOVRay', 'exportScreenshot', 'exportThumbnail') are allowed in the *outputFormats* list.

AJAX data type

```
[{
   String: String,
   String: [Number, Number, String],
   String: String,
   String: Boolean,
   String: String,
   String: Number,
   String: Number | null,
   String: String,
   String: String
 },
 ...]
```

AJAX data description

The data is provided in format:

```
[{
   'groups': <groups>,
   'quality': [<xyres>, <zres>, <pipeline>],
   'format': <format>,
   'compressed': <compressed>,
   'status': <status>,
   'cacheID': <id>,
   'size': <size>,
   'groupsID': <groups id>,
   'CAFSlideUnits': <unit>
 },
 ...]
```

where:

<groups>
is a string describing structures included in the reconstruction,

<xyres>
is the coronal voxel size used in the reconstruction process,

<zres>
is the anterior-posterior voxel size used in the reconstruction process,

<pipeline>
is the service identifier of the pipeline used in the reconstruction process,

<format>
is a string describing the format of reconstruction file,

<compressed>
indicates if the reconstruction file has been compressed as a zip archive,

<status>
is a string describing the status of the reconstruction; possible values are:

- 'None',
- 'Scheduled',
- 'Pending',
- 'In progress',
- 'Completed',
- 'Finished',
- 'Failed',
- 'Removed',

<id>
is the service identifier of the reconstruction,

<size>
is the reconstruction file size (in bytes),

<groups id>
is a string composed of reconstructed groups names separated with '_' character; if an outline is present in the reconstruction, its name precedes others and is separated with double '_' character,

<unit>
is the voxel size unit.

Examples

- http://service.3dbar.org/queryReconstruction?cafDatasetName=sba_DB08;structureList=Br;outputFormats=exportToVRML
- http://service.3dbar.org/queryReconstruction?cafDatasetName=whs_0.6.1;structureList=Brain;outputFormats=exportToVRML,exportToX3d;pipeline=whs_0.6.1-LQ;resolution=0.043,0.043
- http://service.3dbar.org/queryReconstruction?cafDatasetName=sba_DB08;structureList=Amg;switches=brainoutline;outputFormats=exportToVRML,exportToX3d,exportScreenshot

---

INNOVATIVE ECONOMY
NATIONAL COHESION STRATEGY
EUROPEAN UNION
EUROPEAN REGIONAL
DEVELOPMENT FUND

3d Brain Atlas Reconstructor on-line service

getReconstructionByKey

Return reconstruction (stored in the service) of a given service identifier.

syntax

```
getReconstructionByKey?id=<id>
```

Required arguments

id
The service identifier of the reconstruction (a decimal integer).

Example

- http://service.3dbar.org/getReconstructionByKey?id=26721

INNOVATIVE ECONOMY
NATIONAL COHESION STRATEGY

EUROPEAN UNION
EUROPEAN REGIONAL
DEVELOPMENT FUND

3d Brain Atlas Reconstructor on-line service

getThumbnailByKey

Return a thumbnail of reconstruction (stored in the service) of a given service identifier in png format.

syntax

```
getThumbnailByKey?id=<id>
```

Required arguments

id
The service identifier of the reconstruction (a decimal integer).

Example

- http://service.3dbar.org/getThumbnailByKey?id=12064

INNOVATIVE ECONOMY
NATIONAL COHESION STRATEGY
EUROPEAN UNION
EUROPEAN REGIONAL
DEVELOPMENT FUND

3d Brain Atlas Reconstructor on-line service

getPreview

Display window allowing to manipulate (rotate, zoom, etc.) lightweight version of the reconstuciton. Note that the web browser has to support WebGL in order to use this feature.

syntax

```
getPreview?cafDatasetName=<dataset name>
          ;structureName=<structure name>
```

Required arguments

cafDatasetName
The service identifier of the selected CAF dataset.

structureName
Name of structure from hierarchy tree. In practice nearly all names should be fine. Formally name has to point to a root node of a subtree containing at least one node with representation among paths in CAF slides.

Examples

- http://service.3dbar.org/getPreview?cafDatasetName=whs_0.51&structureName=CNS
- http://service.3dbar.org/getPreview?cafDatasetName=whs_0.5&structureName=Hc
- http://service.3dbar.org/getPreview?cafDatasetName=sba_DB08&structureName=FBr

INNOVATIVE ECONOMY
NATIONAL COHESION STRATEGY
EUROPEAN UNION
EUROPEAN REGIONAL DEVELOPMENT FUND

3d Brain Atlas Reconstructor on-line service

getCafInfoPage

Display a window containing basic information about CAF dataset.

syntax

```
getCafInfoPage?cafDatasetName=<dataset name>
```

Required arguments

cafDatasetName
The service identifier of the selected CAF dataset.

Examples

- http://service.3dbar.org/getCafInfoPage?cafDatasetName=sba_DB08

INNOVATIVE ECONOMY
NATIONAL COHESION STRATEGY

EUROPEAN UNION
EUROPEAN REGIONAL
DEVELOPMENT FUND

3d Brain Atlas Reconstructor on-line service

queryJob

Display a window containing information about a reconstruction job of requested identifier

- and about other reconstruction jobs requested for the same CAF dataset.

syntax

```
queryJob?id=<identifier>
```

Required arguments

id
The service identifier of the selected reconstruction job.

Example

- http://www.3dbar.org:8080/queryJob?id=23

INNOVATIVE ECONOMY
NATIONAL COHESION STRATEGY
EUROPEAN UNION
EUROPEAN REGIONAL DEVELOPMENT FUND

How to access the *Custom Reconstruction Wizard?*

Registered users are able to perform and manage custom reconstructions. Custom reconstructions can be submitted using the *Custom Reconstruction Wizard*. After logging in, the wizard can be accessed in two ways:

- From the **Live Preview** tab,
- From the **Reconstructions** tab.

Accessing from the *Live Preview* tab

1. Make sure that you are logged in. When you are not logged in, the custom reconstruction features are not available.
2. Select desired structures for reconstruction (at least one structure has to be selected). Note that when no structures are selected, the **Get custom reconstruction** button is not visible.
3. Structures selected in **Live preview** tab are automatically transferred to the reconstruction wizard (see screenshot below).

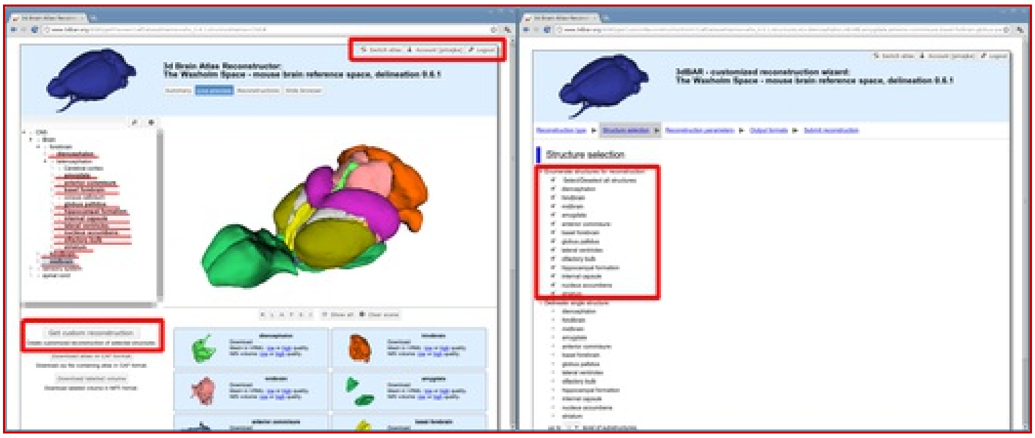

You have to be logged in in order to access the *Custom Reconstruction Wizzard. Structures loaded in* Live preview *window are automatically transferred to the reconstruction wizard.*

Accessing from the *Reconstructions* tab

1. Make sure that you are logged in. When you are not logged in, the custom reconstruction features are not available.
2. Type the structure name into **Search for the following structure** box,
3. Hit the **Search for reconstructions** button,
4. At the top of the table containing search results you can find a link that allows you to perform a custom reconstruction of structure that you searched for.

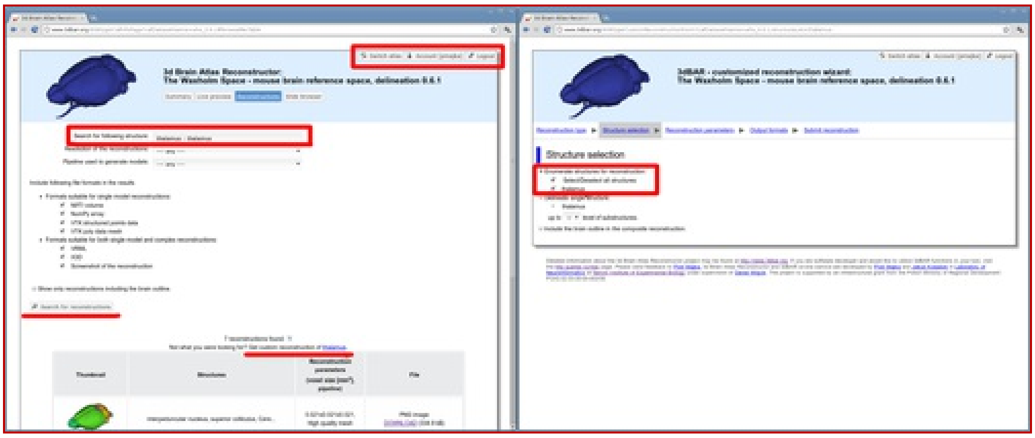

Accessing the reconstruction wizzard from the *Reconstructions* tab.

INNOVATIVE ECONOMY
NATIONAL COHESION STRATEGY

EUROPEAN UNION
EUROPEAN REGIONAL DEVELOPMENT FUND

How to use labeled (indexed) volumes?

How to use labeled volumes available at 3dBAR service website?

You can download two types of volumetric datasets from 3dBAR service. One of them is labeled volume containing all structures from given atlas. If such volume is available for a chosen atlas, you can download it either from **Live Preview** or **Atlas Details** tab.

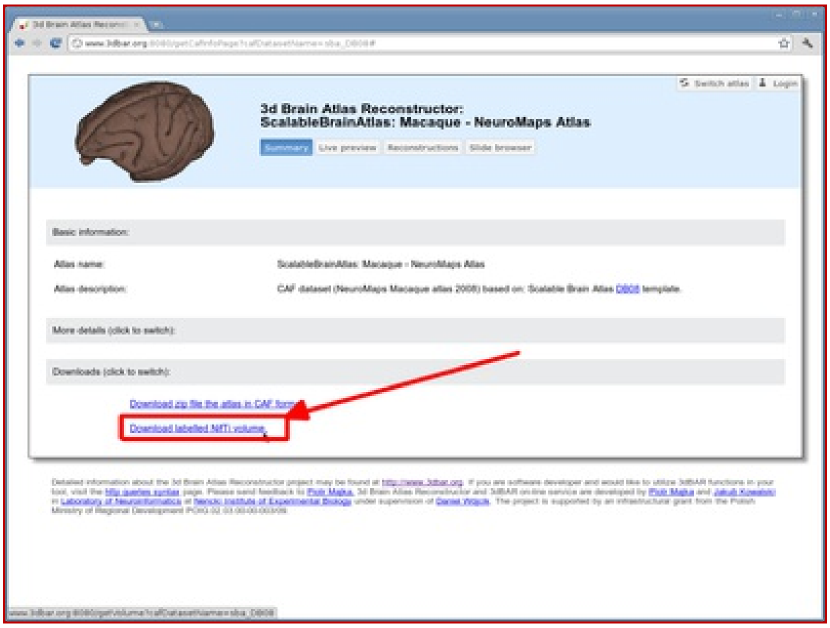

Downloading labeled volume from 'Atlas Details' tab

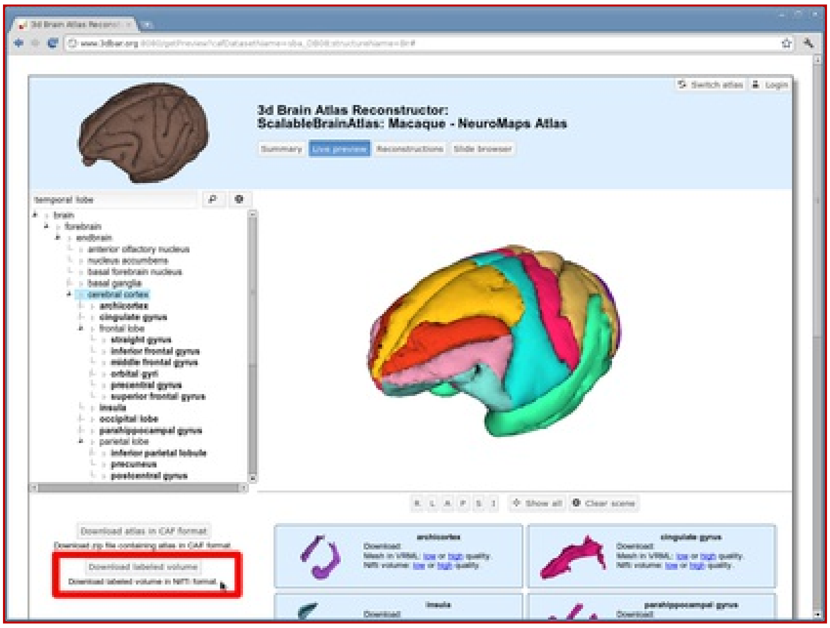

Downloading labeled volume from 'Live preview' tab

The tutorial below demonstrates how to load the labeled volumes along with the appropriate lookup table into ITK-Snap and how to generate polygonal meshes of selected structures. Similar tasks may be performed using other software (e.g. Paraview or 3D Slicer), we have choosen ITK-Snap as we find it lightweight and convenient to use. As an example, we will use the indexed volume of NeuroMaps Macaque monkey brain atlas available from Scalable Brain Atlas.

Steps to follow:

1. Download a zip file with the labeled volume from 3dBAR website and extract its contents. The zipfile contains three files:
    - The indexed volume itself (*_indexed_volume.nii.gz),
    - A lookup table providing names of structures along with a color map (*_lut.txt),
    - A text file containing basic description of the atlas and the licensing information (*_3dbar-info.txt).

2. Open ITK-Snap and select **Open Grayscale Image...** from the **File** menu. Then, select the NIfTI volume you extracted. The volume will be loaded as a grayscale image.

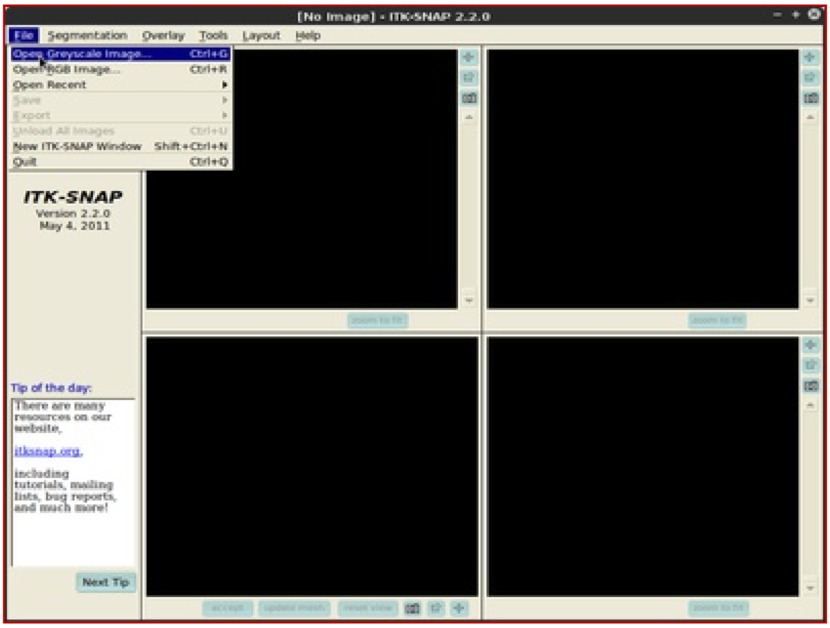

Open Grayscale Image…

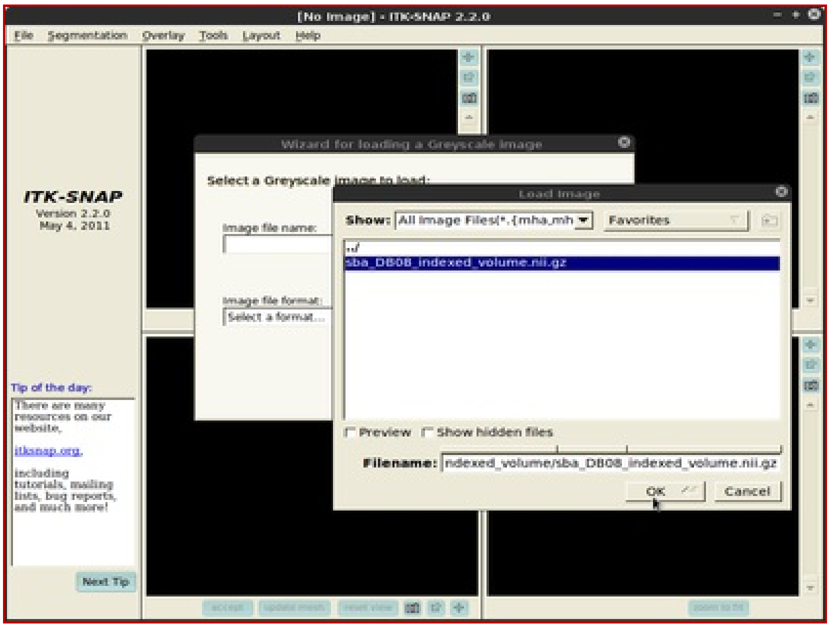

Choose NIfTI volume you extracted

3. Then, choose **Segmentation**, **Load from image** menu and select the same volume file as in the previous point. This time the volume will be loaded as *Segmentation* denoting consecutive structures.

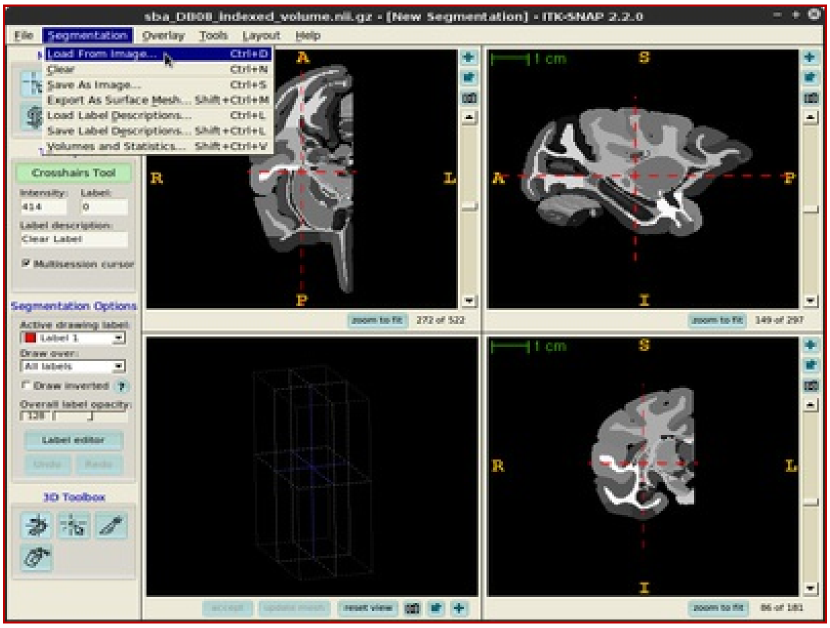

Choose 'Segmentation', then 'Load from image'

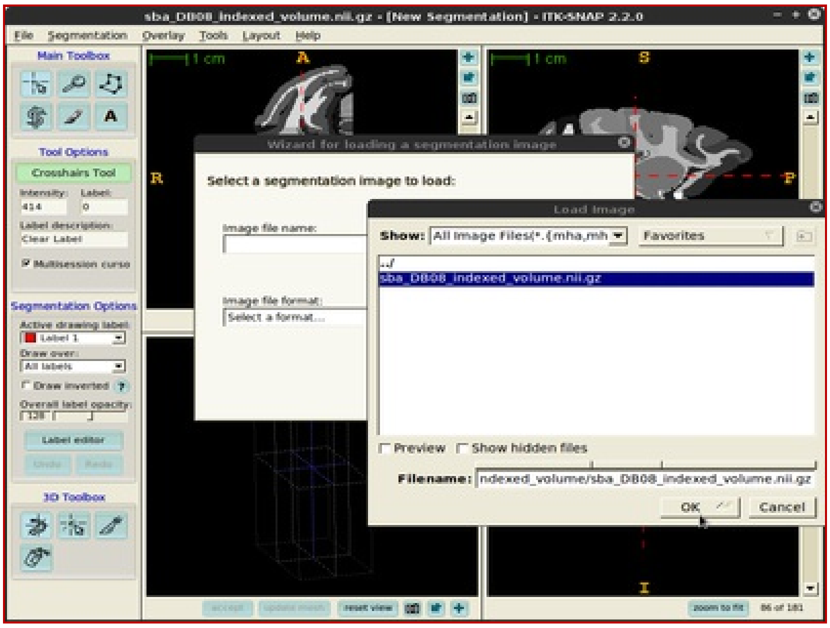

Select the same NIfTI file

4. The next task is to load the lookup table. From the **Segmentation** menu select **Load Label Descriptions...** and select the text file with the lookup table. Now all indexed structures from the *Segmentation* image are linked with appropriate names and segmentation colors.

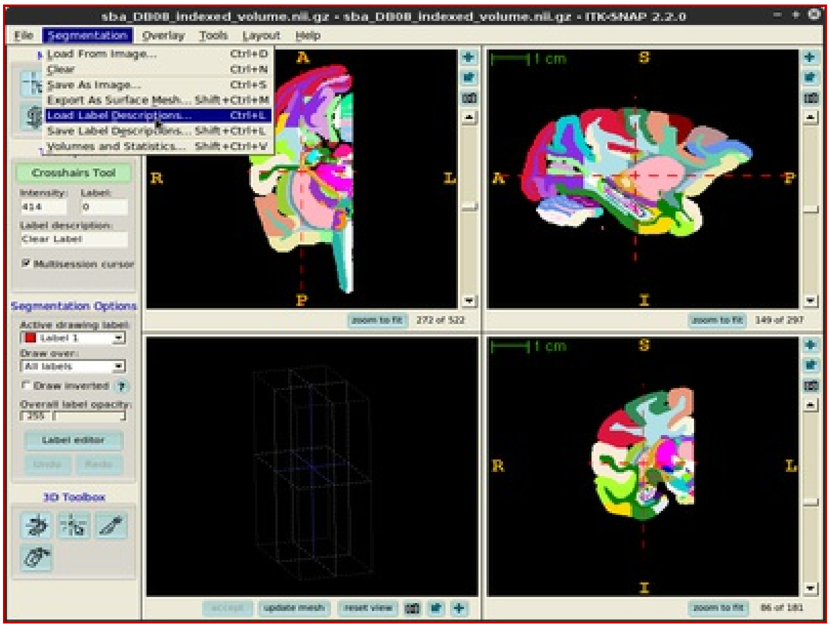

"Segmentation", "Load Label Descriptions..."

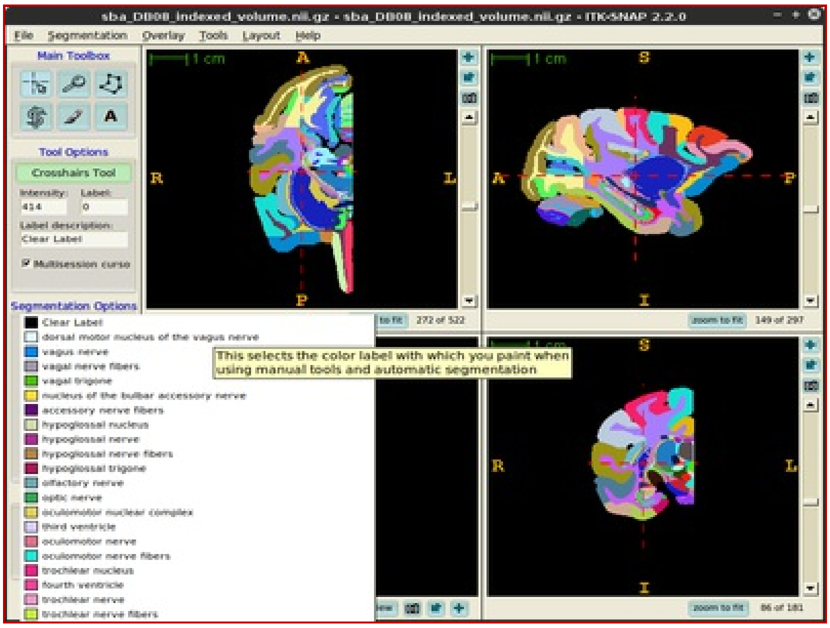

5. In order to generate a surface mesh click **Update mesh** button located at the bottom of the ITK-Snap window. After a while the mesh will be ready.

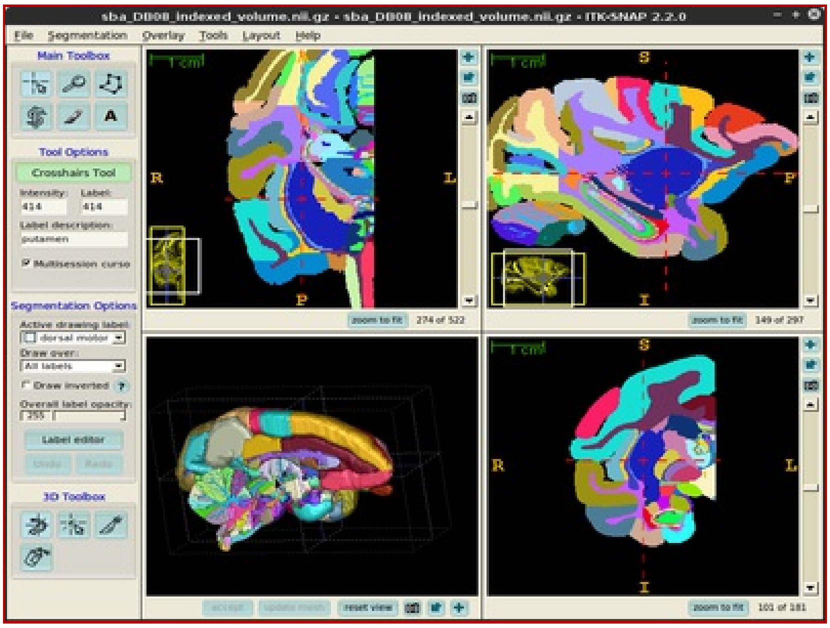

Click 'update mesh' button

6. You can also export the surface meshes for selected structures (single structure as well as multiple structures in a single file). In order to do this, choose **Segmentation**, **Export As Surface Mesh** and follow the on-screen instructions provided by the **3D Mesh Export Wizard**.

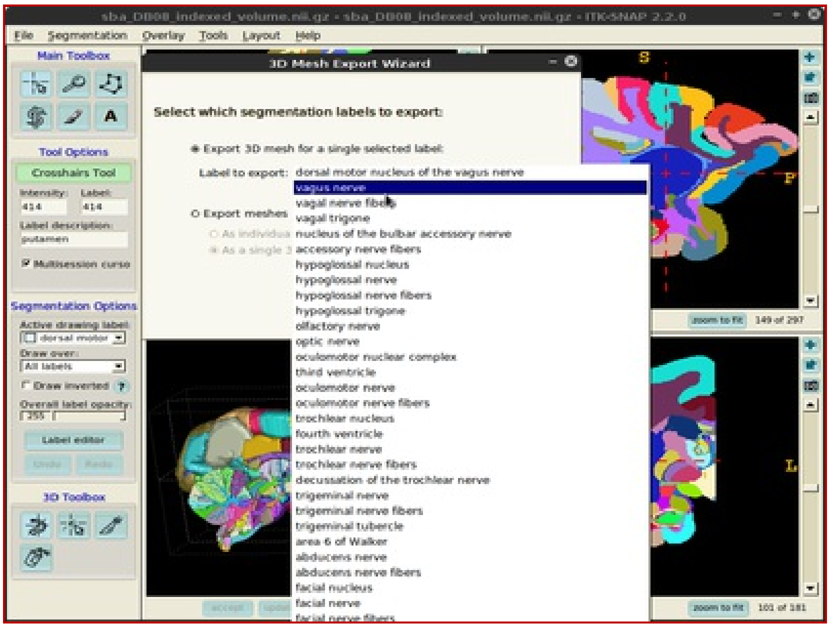

"Segmentation", "Export As Surface Mesh"

7. You can always customize surface mesh generation settings using **Tools** -> **Display Options** -> **3D Rendering** dialogue.

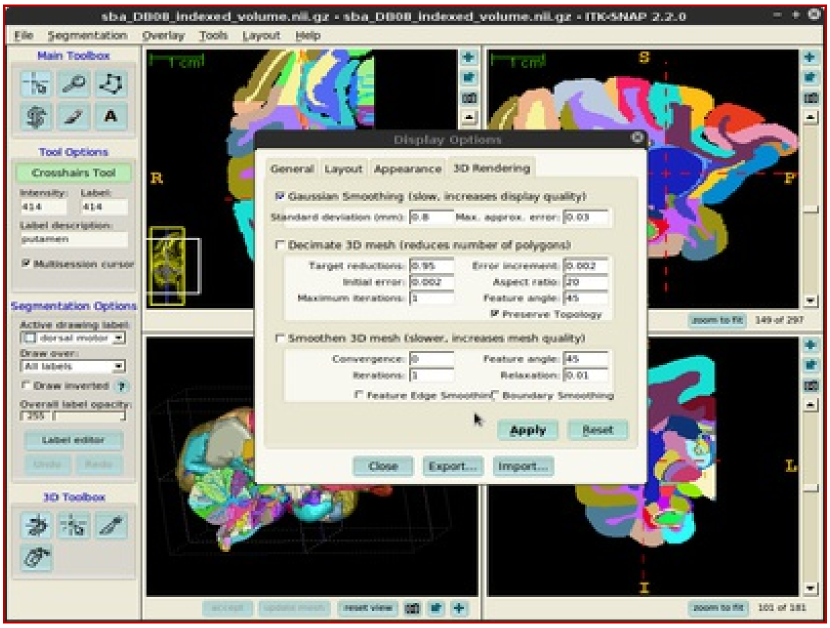

INNOVATIVE ECONOMY
NATIONAL COHESION STRATEGY

EUROPEAN UNION
EUROPEAN REGIONAL DEVELOPMENT FUND

Troubleshooting

At the moment we are supporting only Chrome browser. The reason is that it is free, multiplatform, popular and widely-available, so every user of a modern computer can use it. Moreover we use it developing the service so many (at the moment all) issues are reported by ourself.

You can help us to support your browser. If you figure out how to solve your issue, please let us know about solution so we can append it to this document. Good idea is to begin with inspecting available solutions - they might work with your browser too (or at least give you a clue what the nature of the issue might be).

Issues

- Live preview does not work in my browser

INNOVATIVE ECONOMY
NATIONAL COHESION STRATEGY

EUROPEAN UNION
EUROPEAN REGIONAL
DEVELOPMENT FUND

Chrome

If you are Linux user, then (according to http://www.borfast.com/blog/how-enable-webgl-google-chrome-linux-blacklisted-graphics-card) you can try running it from commandline with *--enable-webgl* and *--ignore-gpu-blacklist* switches:

```
$ google-chrome --enable-webgl --ignore-gpu-blacklist
```

It should be working for other operating systems too. Moreover, you can set appropriate flags with chrome://flags/ URL in your Chrome browser.

You can be also interested in visiting:

- for Mac OS:
  - Does WebGL work on Chrome on a Mac?
  - How to use software webgl engine in Chrome 18
- for Android:
  - Google finally makes it easy to enable WebGL support in latest Chrome for Android beta
- for Windows:
  - WebGL doesn't work under Chrome in Windows 8 Developer Preview
  - How do I enable WebGL in Google Chrome 11? I'm running it on Windows XP
- generic:
  - http://get.webgl.org/
  - http://en.wikipedia.org/wiki/WebGL
  - WebGL and 3D graphics
  - GPU accelerating 2D Canvas and enabling 3D content for older GPUs

Other browsers

You can help us to support your browser. If you figure out how to solve your issue, please let us know about solution so we can append it to this document. Good idea is to begin with inspecting solutions available for Chrome - they might work (or give you a clue what the nature of the issue might be).

You might be also interested in visiting:

- http://get.webgl.org/
- http://en.wikipedia.org/wiki/WebGL

INNOVATIVE ECONOMY
NATIONAL COHESION STRATEGY
EUROPEAN UNION
EUROPEAN REGIONAL DEVELOPMENT FUND

Table of Content

Table of Content 32
Software we recommend to use with 3dBAR service data 33
Using atlas data as a spatial reference for a result of a realistic neural network simulation 35
Model download 35
Visualisation 35
Attachments 38
WebGL Issue 39
Browsers' compatibility with the service 39
Chrome 40
Other browsers 40

Software we recommend to use with 3dBAR service data

Software	Suitable file formats
3dBAR	CAF
3D Slicer	• NIfTI,<br>• STL (STereoLithography),<br>• VTK structured grid,<br>• VTK polydata
BioImage Suite	• NIfTI,<br>• VTK structured grid
ITK-SNAP	• NIfTI,<br>• VTK structured grid
ParaView	• STL (STereoLithography),<br>• VRML,<br>• VTK polydata,<br>• VTK structured grid,<br>• NIfTI
Blender	• STL (STereoLithography),<br>• VRML,<br>• X3D,<br>• SVG
NumPy	NumPy array
Inkscape	• SVG,<br>• PNG
Gimp	PNG

File format	Recommended software
CAF	3dBAR
NIfTI	• ParaView v. 3.14.1,<br>• ITK-SNAP,<br>• BioImage Suite,<br>• 3D Slicer
NumPy array	NumPy
PNG	• Gimp
STL (STereoLithography)	• ParaView,<br>• Blender,<br>• 3D Slicer
SVG	Inkscape
VRML	• ParaView,<br>• Blender
VTK polydata	• ParaView,<br>• 3D Slicer
VTK structured grid	• ParaView v. 3.14.1,<br>• ITK-SNAP,<br>• BioImage Suite,<br>• 3D Slicer
X3D	Blender

INNOVATIVE ECONOMY
NATIONAL COHESION STRATEGY
EUROPEAN UNION
EUROPEAN REGIONAL DEVELOPMENT FUND

Using atlas data as a spatial reference for a result of a realistic neural network simulation

Model download

From The Allen Mouse Brain Reference Atlas, 2011 Segmentation download the following VRML high quality models:

- Basic cell groups and regions,
- Primary somatosensory area, barrel field, layer 1,
- Primary somatosensory area, barrel field, layer 2/3,
- Primary somatosensory area, barrel field, layer 4,
- Primary somatosensory area, barrel field, layer 5,
- Primary somatosensory area, barrel field, layer 6a,
- Primary somatosensory area, barrel field, layer 6b,
- Ventral posterolateral nucleus of the thalamus.

Download also a model of a of the barrel cortex column. Unwrap downloaded archives.

Visualisation

Run ParaView software (description for version 4.0.1). Open downloaded *.wrl and *.vtk files. Click the *Apply* button (in the tab *Properties*).

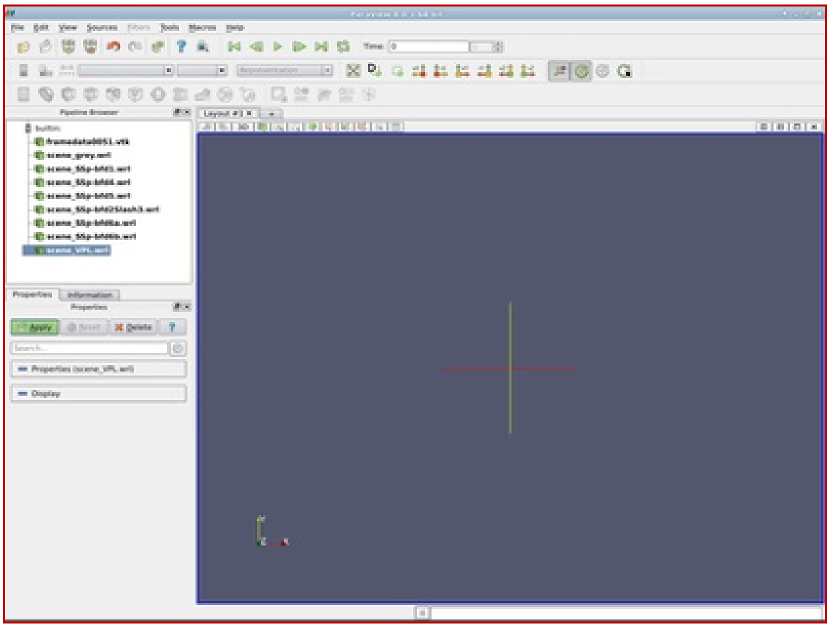

Loaded models.

In the *Properties* tab set *Styling: Opacity* to 0.1 for *scene_grey.wrl* model. For every *scene_SSp_bfd*.wrl* model set *Styling: Opacity* to 0.3, then change *Coloring* from *VRMLColor* to *Solid Color*. Click *Coloring: Edit* and select color to:

- Red = 250, Green: 245, Blue: 255 for *scene_SSp_bfd1.wrl*,
- Red = 153, Green: 163, Blue: 255 for *scene_SSp_bfd2Slash3.wrl*,
- Red = 74, Green: 77, Blue: 128 for *scene_SSp_bfd4.wrl*,
- Red = 171, Green: 177, Blue: 255 for *scene_SSp_bfd5.wrl*,
- Red = 145, Green: 149, Blue: 213 for *scene_SSp_bfd6a.wrl*,
- Red = 177, Green: 170, Blue: 255 for *scene_SSp_bfd6b.wrl*.

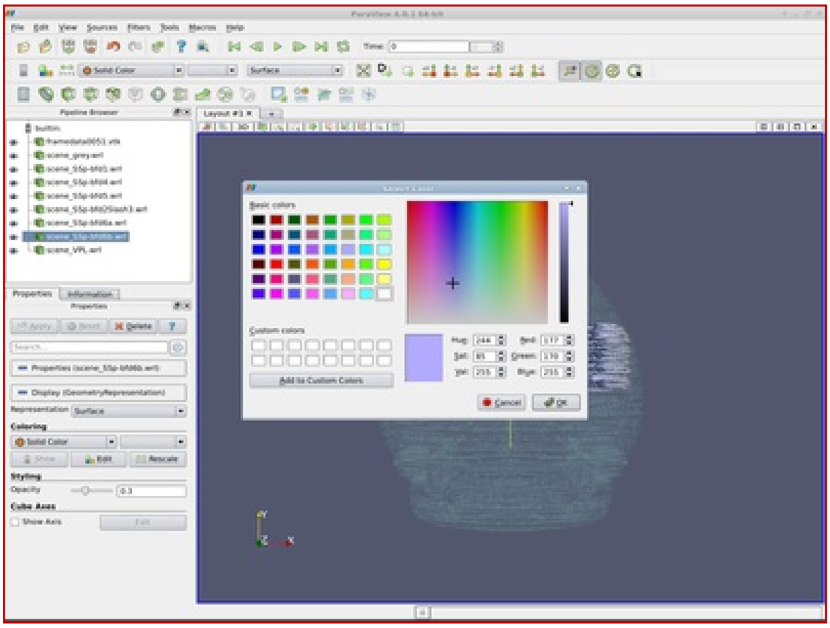

Color and opacity settings.

Click *Coloring: Edit* for the *framedata0051.vtk* model (ensure that *Coloring* is set to *voltage*). Click the "Gear" icon to edit *Color Scale*. Set *Color Space* to *CIELAB*; set the left color point to Red = 59, Green = 76 and Blue = 192 and the right to Red = 255, Green = 255 and Blue = 0. Add two color points: at *Color Scalar Value* = -40 (Red = 150, Green = 150 and Blue = 0) and at *Color Scalar Value* = 18 (Red = 190, Green = 35 and Blue = 21). Click the *Apply* button, then close the *Color Scale Editor* window.

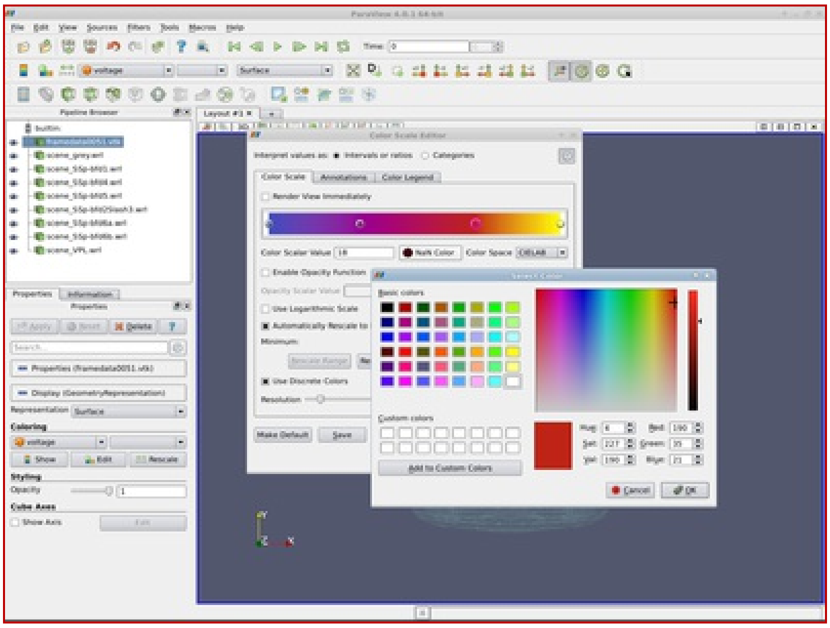

Voltage to color mapping.

Apply the *Transform* filter (*Filters/Alphabetical/Transform* from menu) to the *framedata0051.vtk* model. In the *Properties* tab set the transformation matrix to:

Translate	-3.71011222757522	-0.955791920040867	-0.855289018330382
Rotate	40.2025580192141	-63.976105129058	76.2999549515704
Scale	0.58	0.58	0.58

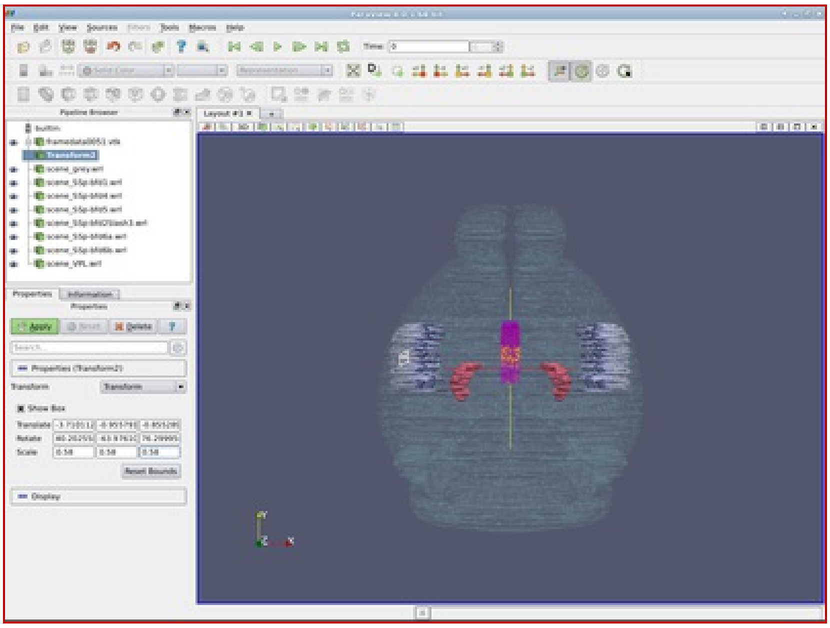

The transformation filter.

Click the *Apply* button.

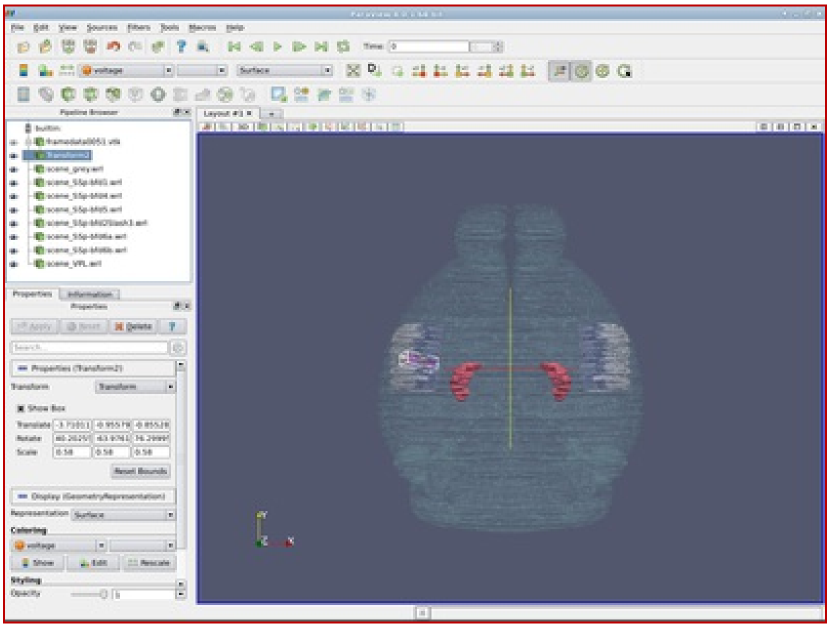

A complete scene.

Choose an appropriate viewport and export the scene or a screenshot if you wish.

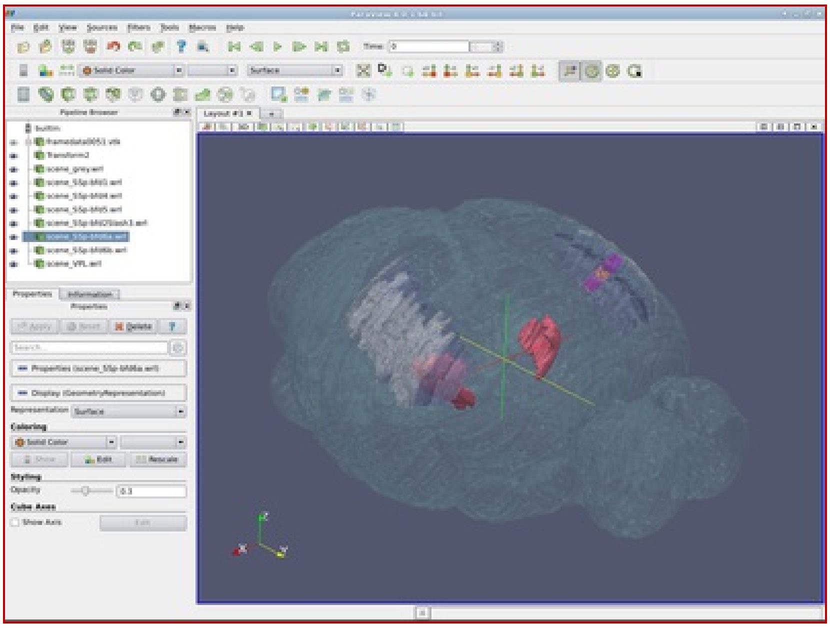

The complete scene at another viewpoint.

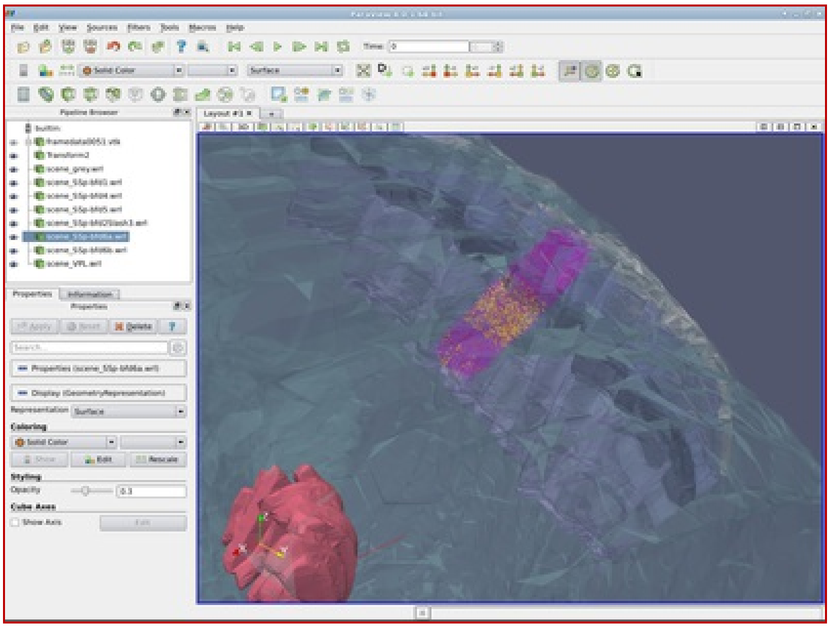

The complete scene - focus on the model of barrel cortex column.

▶ Attachments

INNOVATIVE ECONOMY
NATIONAL COHESION STRATEGY

EUROPEAN UNION
EUROPEAN REGIONAL DEVELOPMENT FUND

WebGL Issue

Browsers' compatibility with the service

browser	Linux	Windows 7	OS X
Google Chrome	23.0.1271.97	23.0.1271.64 m	27.0.1453.116
Chromium	25.0.1364.160	Not tested	Not tested
Safari	Not tested	Not compatible	6.0.5
Mozilla Firefox	21.0	21.0	21.0
Opera	Not compatible	Not compatible	Not tested
Konqueror	Not compatible	Not tested	Not tested
Internet Explorer	Not tested	Not compatible	Not tested
Links	Not compatible	Not tested	Not tested

Key to the background color			
The browser has not been tested under given operating system.	The tested version of browser is fully compatible with the service.	The tested version of browser is not fully compatible with the service.	No compatible version of browser has been found

Chrome

If you are Linux user, then (according to http://www.borfast.com/blog/how-enable-webgl-google-chrome-linux-blacklisted-graphics-card) you can try running it from commandline with *--enable-webgl* and *--ignore-gpu-blacklist* switches:

```
$ google-chrome --enable-webgl --ignore-gpu-blacklist
```

It should be working for other operating systems too. Moreover, you can set appropriate flags with chrome://flags/ URL in your Chrome browser.

You can be also interested in visiting:

- for Mac OS:
  - Does WebGL work on Chrome on a Mac?
  - How to use software webgl engine in Chrome 18
- for Android:
  - Google finally makes it easy to enable WebGL support in latest Chrome for Android beta
- for Windows:
  - WebGL doesn't work under Chrome in Windows 8 Developer Preview
  - How do I enable WebGL in Google Chrome 11? I'm running it on Windows XP
- generic:
  - http://get.webgl.org/
  - http://en.wikipedia.org/wiki/WebGL
  - WebGL and 3D graphics
  - GPU accelerating 2D Canvas and enabling 3D content for older GPUs

Other browsers

You can help us to support your browser. If you figure out how to solve your issue, please let us know about solution so we can append it to this document. Good idea is to begin with inspecting solutions available for Chrome - they might work (or give you a clue what the nature of the issue might be).

You might be also interested in visiting:

- http://get.webgl.org/
- http://en.wikipedia.org/wiki/WebGL

INNOVATIVE ECONOMY
NATIONAL COHESION STRATEGY
EUROPEAN UNION
EUROPEAN REGIONAL DEVELOPMENT FUND
